# A Systematic Review of Machine Learning and IoT Applied to the Prediction and Monitoring of Cardiovascular Diseases

**DOI:** 10.3390/healthcare11162240

**Published:** 2023-08-09

**Authors:** Alejandra Cuevas-Chávez, Yasmín Hernández, Javier Ortiz-Hernandez, Eduardo Sánchez-Jiménez, Gilberto Ochoa-Ruiz, Joaquín Pérez, Gabriel González-Serna

**Affiliations:** 1 Computer Science Department, Tecnológico Nacional de México/Cenidet, Cuernavaca 62490, Mexico; javier.oh@cenidet.tecnm.mx (J.O.-H.); m22ce005@cenidet.tecnm.mx (E.S.-J.); joaquin.po@cenidet.tecnm.mx (J.P.); gabriel.gs@cenidet.tecnm.mx (G.G.-S.); 2 School of Engineering and Sciences, Tecnologico de Monterrey, Av. Eugenio Garza Sada 2501, Monterrey 64849, Mexico; gilberto.ochoa@tec.mx

**Keywords:** systematic review, cardiovascular disease, machine learning, wearable technologies, IoT, IoMT

## Abstract

According to the Pan American Health Organization, cardiovascular disease is the leading cause of death worldwide, claiming an estimated 17.9 million lives each year. This paper presents a systematic review to highlight the use of IoT, IoMT, and machine learning to detect, predict, or monitor cardiovascular disease. We had a final sample of 164 high-impact journal papers, focusing on two categories: cardiovascular disease detection using IoT/IoMT technologies and cardiovascular disease using machine learning techniques. For the first category, we found 82 proposals, while for the second, we found 85 proposals. The research highlights list of IoT/IoMT technologies, machine learning techniques, datasets, and the most discussed cardiovascular diseases. Neural networks have been popularly used, achieving an accuracy of over 90%, followed by random forest, XGBoost, k-NN, and SVM. Based on the results, we conclude that IoT/IoMT technologies can predict cardiovascular diseases in real time, ensemble techniques obtained one of the best performances in the accuracy metric, and hypertension and arrhythmia were the most discussed diseases. Finally, we identified the lack of public data as one of the main obstacles for machine learning approaches for cardiovascular disease prediction.

## 1. Introduction

According to the Pan American Health Organization, cardiovascular disease (CVD) is the leading cause of death worldwide, claiming an estimated 17.9 million lives each year [1]. CVD refers to a group of diseases affecting the heart and blood vessels, including coronary heart disease (acute myocardial infarction), cerebrovascular disease, peripheral arterial disease, congenital heart disease, rheumatic heart disease, and venous and pulmonary thrombosis [2]. The major behavioral risk factors for heart disease and stroke are physical inactivity, harmful use of alcohol, unhealthy diet, and tobacco use [3]. More than four out of five CVD deaths are due to strokes and heart attacks, and one-third of these deaths occur prematurely in people under the age of 70 [3]. The World Health Organization projects that nearly 23.6 million people will die from CVD by 2030, and it is predicted to remain the leading cause of death worldwide [2]. Low- and middle-income countries account for more than 75% of CVD deaths [2]. Thus, the analysis of mortality due to CVD globally has become a top priority.

Artificial intelligence technologies are rapidly growing, and IoT and machine learning approaches can now be used to monitor and even predict CVD. The IoT consists of everyday objects connected to the Internet without human interaction [4]. The application of the tools, principles, concepts, and techniques used in the accepted Internet of Things approach within the medical and healthcare sectors is called the Internet of Medical Things (IoMT) [5]. Machine learning, on the other hand, is defined as a branch of artificial intelligence techniques that extract knowledge from data, also known as predictive analytics or statistical learning [6]. In particular, these techniques derive models from data (i.e., information such as documents, audio, and images), where the resulting model is the final product of machine learning [7]. Machine learning applied to medicine can transform existing modes of healthcare delivery [8].

Technological solutions based on IoMT and machine learning can improve the quality of life of patients diagnosed with CVD by preventing risk conditions, as well as helping those without easy access to healthcare services. The use of IoMT through smart devices enables the real-time detection, monitoring, and prediction of CVD, as well as allowing for emergency communication (i.e., alerts sent to caregivers or hospitals), all integrated into a portable device. Over the past few years, IoT/IoMT has evolved rapidly, with sensors or devices that are more powerful and capable of monitoring the vital signs of patients with chronic diseases, making it one of the most important technologies. The integration of smart wearables through IoT/IoMT has a significant impact on modern healthcare systems, providing value to health-seekers, delivering high-quality and cost-effective services, and facilitating effective remote care. However, the amount of data generated by these devices in the cloud environment is a major concern, which has led to several challenges, including determining the best machine learning techniques to mine this data. Many applications and frameworks have been developed using machine learning and deep learning for CVD prediction and monitoring, improving the quality of healthcare and providing accurate results. Thus, it is important to be aware of and analyze the state-of-the-art of the techniques and technologies being implemented to predict, monitor, or classify CVD.

We conducted a systematic review to highlight the use of IoT/IoMT and machine learning in the detection, prediction, or monitoring of CVD. We adopted the PRISMA [9] guidelines (Preferred Reporting Items for Systematic Reviews) and the research scope was based on the application of the PICOC framework [10]. This systematic review aims to identify state-of-the-art CVD and machine learning approaches based on four main contributions: (i) IoT/IoMT devices for monitoring or predicting cardiovascular disease; (ii) different types of CVD in the population; (iii) machine learning applications for detecting, predicting, or monitoring CVD; and (iv) data sets used with such machine learning techniques.

The main goal of this paper is to identify the current state of IoT/IoMT in CVD detection or monitoring, machine learning techniques, and data sets used to predict or classify these conditions. Despite the existence of literature reviews on the use of IoT/IoMT technologies to detect, predict, or monitor CVD, a compilation of research proposals on the use of these technologies in combination with machine learning techniques and the data sets used remains lacking. We aim to collectively analyze data sets, IoT/IoMT wearable devices/smart devices/medical devices, machine learning approaches, and disease types. In this paper, we present the best-practice technological devices, relevant machine learning approaches, evaluation metrics, and their results over the past seven years.

**Figure 1:**
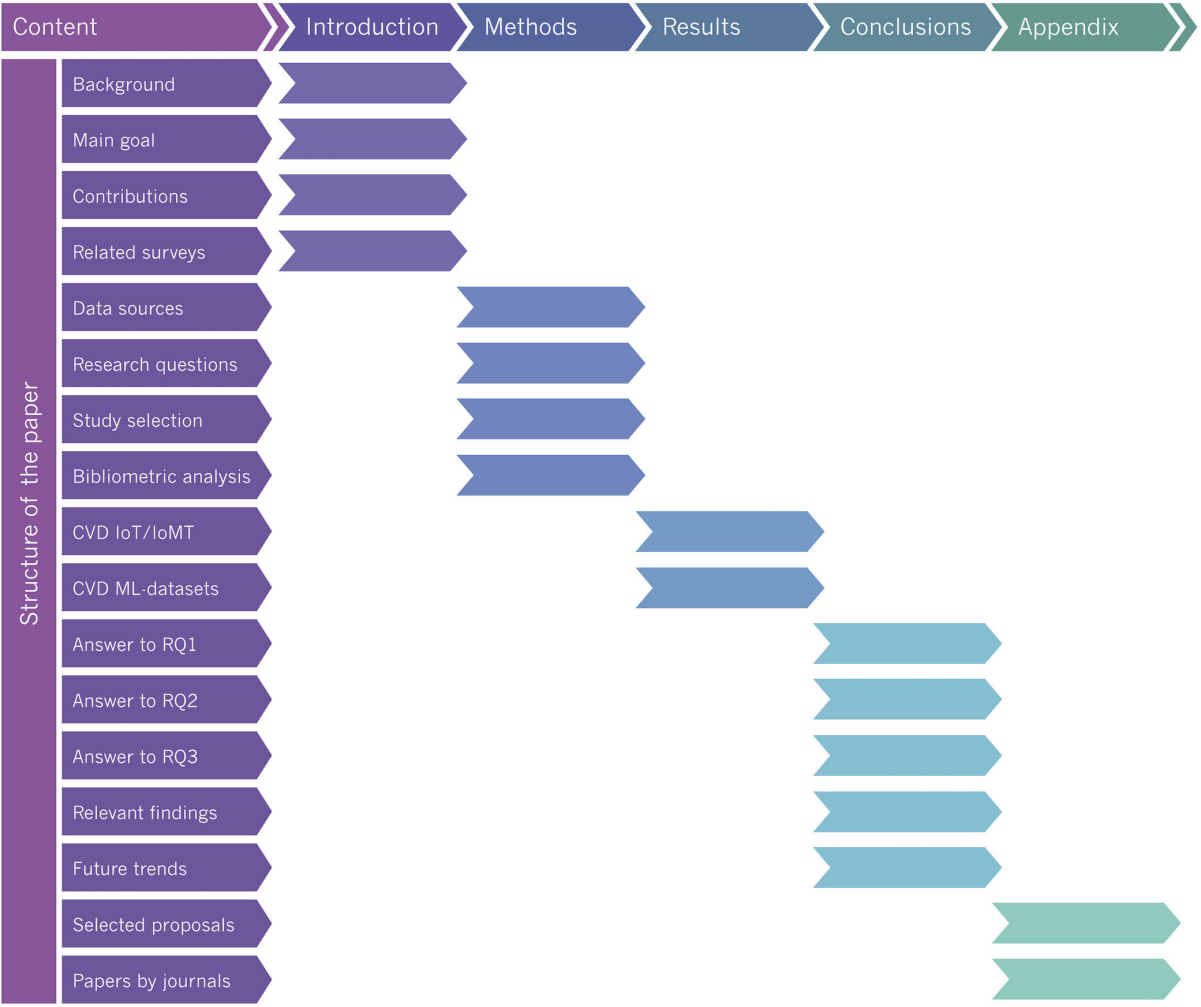
Structure of the paper as a comprehensive study roadmap.

The remainder of this paper is organized as follows: Section 2 describes the related surveys. Section 3 presents the methods, such as the research question, the scope of the study, the literature review, and the inclusion and exclusion criteria. Section 4 describes the selected papers using IoT/IoMT technology. Section 5 presents the selected papers applying machine learning techniques. Section 6 discusses the papers considered for further analysis. Finally, Section 7 concludes the paper with future directions. The structure of the paper, as a comprehensive study roadmap, is shown in Figure 1.

## 2. Related Surveys

Several articles have been published on IoT/IoMT technologies and machine learning techniques in healthcare. This section briefly reviews the studies detailing systematic reviews on IoT/IoMT technologies applied to CVD along with machine learning methods and their results. We found that some of these articles focused only on the machine learning approaches and their results in a specific disease, while others focused on the technologies applied for disease monitoring.

For example, along with some recommendations, Friedrich et al. [11] focused on applications of machine learning approaches related to cardiovascular drugs. They identified 215 studies in their systematic review using PubMed and Embase as research databases. They concluded that 87% of the methods used belong to the supervised learning context (tree-based methods being the most common, followed by network and regression analysis, and boosting approaches). Similarly, Hazra et al. [12] provided brief descriptions of 35 research papers published between 2006 and 2016 which examined computational methods for predicting heart disease. They concluded that, among the classification techniques, Decision Tree, Naïve Bayes, Artificial Neural Networks, Association Rule Mining, and Fuzzy Logic were the most commonly used. The data mining tools with better results—in terms of practical execution—were Java, WEKA, Tanagra, and Matlab. On the other hand, Shameer et al. [13] discussed machine learning algorithms and potential data sources. They summarized the open-access biomedical and healthcare ontologies and big data resources in cardiovascular medicine for the development of machine learning resources. Furthermore, they assessed the potential limitations and challenges associated to implementing AI in medicine. Bolhasani et al. [14] explored deep learning techniques for healthcare IoT applications. They presented how deep learning can address telemedicine and ambient assisted living systems, machine health monitoring systems, human activity recognition, patient vital signs collection, and data fusion. Their survey divided the research studies into four categories: Medical diagnosis and differentiation applications, home and personal health applications, disease prediction applications, and human behavior recognition applications. They identified the number of studies by deep learning techniques used (convolutional neural networks being the most-used) and the proposed evaluation environments (data set, implementation, and simulation).

Other papers have focused on IoT/IoMT technologies applied in healthcare systems or applications. Huang et al. [15] focused on IoT technologies for health management system—including clinical device management, medication management, clinical data management, remote medicine, mobile medical care, and individual health management—with the purpose of serving as a starting point for future IoT/IoMT security management and design. Lin et al. [16] presented recent developments in monitoring various physiological signals using flexible sensors for CVD through pulse wave technology (ECG, PCG, PPG, and SCG/BCG); in particular, they focused on five types of signals that can reflect CVD using flexible sensing technology. Rahaman et al. [17] presented IoT-based smart health monitoring systems with their advantages and disadvantages, highlighting the design and implementation of these monitoring devices with respect to the patients. They summarized 13 studies from 2015 to 2019, including the year, feedback device, key hardware components, use, and cost. Panicker and P. Gayathri [18] classified their work into various categories, such as feature selection, heart sounds, heart images, heart rate variability, IoT/wearable technology, fuzzy systems, and predictive models. They presented a tabulated summary in their literature review highlighting the different machine learning techniques and data sets used, results, and research gaps. However, this summary included only 13 works published between the years 2015 and 2018. The remaining works were cited and described in the document.

Other papers have discussed relevant studies focused on chronic diseases such as diabetes, cancer, CVD, hypertension, and glaucoma. Dadkhah et al. [19] summarized the use of IoT for chronic disease management, and concluded that CVD is one of the highest priorities for the use of IoT in the context of developing countries. Their results included 92 studies classified into 47 focused on CVD, 37 on hypertension, 5 on cerebrovascular, 1 on rheumatic, 1 on rheumatism, and 1 on ischemic. Lamonaca et al. [20] focused on monitoring blood pressure from a metrological point of view, aiming to address the lack of traceability and reliability of BP measurements. They analyzed the vulnerabilities and opportunities of smart devices and wearables, including medical devices, in terms of accuracy and reliability. They focused on smart metering devices, Internet-connected devices, and devices that enable the implementation of the Internet of Medical Things (IoMT).

Since 2020, reviews have focused on artificial intelligence for disease diagnosis, highlighting the analysis of cardiovascular disease. Argha, Celler, and Lovell [21] reviewed AI-based blood pressure estimation approaches with a focus on recent advances in deep learning-based techniques. They concluded that deep learning methods make it possible to develop reliable and accurate blood pressure estimation algorithms/devices. They also noted the lack of adequate data sets on invasive and non-invasive blood pressure as standard references. Huang et al. [22] identified and described recent developments in the application of digital health to CVD, focusing on AI models driven by data collected from wearables. They reported the type of disease detected, the algorithms applied, the application, and the performance for machine learning and deep learning approaches. Hinai et al. [23] identified articles on the end-to-end deep learning analysis of resting ECG signals for the detection of structural cardiac pathology. They identified 12 articles, 3 of which detected left ventricular systolic dysfunction, 1 of which detected left ventricular hypertrophy, 6 of which detected acute myocardial infarction, and 2 of which detected stable ischemic heart disease. The performance measures used were AUC and accuracy. On the other hand, Faizal et al. [24] outlined various conventional models for assessing and predicting risk and compared them with AI-based approaches. They briefly reported some deep learning and machine learning algorithms, with their respective performance, focusing on the country, study area, risk factors, and performance.

Chen et al. [25] reported on the use of deep learning algorithms in medical technology applications, focusing on challenges and recommendations for ECG detection and classification. The highlights of this paper were as follows: algorithms for CVD detection and classification, smart wearable devices and hardware based on deep learning and ECG, and recognition using ECG biological signs. Qureshi et al. [26] presented ambient assisted living solutions to reduce morbidity and mortality in patients with cardiovascular conditions. They focused on the application, devices, testing platform, monitored signals, features, and limitations for ambient assisted solutions and monitoring/clinical management. They also focused on the purpose, architecture, accuracy, deep learning methods, databases, and pre-processing of deep learning-based solutions for ambient assisted living. However, they only reviewed articles published between 2015 and 2019, and selected only 40 as relevant to their research. Bhushan, Pandit, and Garg [27] discussed how machine learning and deep learning approaches have been used for the analysis of various heart diseases. They classified the articles by summary, technique/tool, advantages, disadvantages, and performance measure. The existing works related to ensemble models using machine learning and deep learning, as well as a description of relevant data sets, were reported as a separate section.

The review of Rath et al. [28] included methods for feature extraction, selection, and reduction, as well as machine learning-/deep learning-based classification schemes, CVD data sets, and types of heart disease. They also listed some heart disease attributes identified in the 60 collected papers. Maurya et al. [29] reviewed studies on the early prediction of heart failure, determination of its severity, prediction of adverse outcomes, and improving patient adherence to medication. They focused on the parameters measured, endpoint, impact on heart failure, algorithms, evaluation measures, and the data (e.g., how patients were monitored, data sets). However, they did not explain the study selection, whether they used a methodology or guidelines for the review, or how many papers they found relevant. Kumar et al. [30] conducted a survey based on artificial intelligence to diagnose chronic disease including heart disease, stroke, and hypertension. They focused on healthcare applications, type of disease, data set, technique, reported outcomes, feature extraction, and the classification process for prediction.

Jasinska-Piadlo et al. [31] offered a comprehensive examination of the utilization of data science and machine learning in heart failure data sets. They summarized significant discoveries while critically assessing the effectiveness, suitability, and precision of various approaches. In different sections, they also reported the dimensionality of the used data sets, missing data, the performance of the algorithms (for the most-used machine learning methods) and, most importantly, how machine learning and data analysis impact heart failure problem-solving. Chakrabarti et al. [32] reported the diagnostic applications of wrist-worn devices in detecting multiple diseases, including cardiovascular conditions. They also provided a brief discussion of machine learning algorithms for wearable data analysis and addressed the current challenges associated with wearables and medical data. Finally, Guo et al. [33] reviewed the advancements in wearable devices—specifically, unobtrusive sensing technologies—that provide support and tools for the management of chronic disease. They not only focused on cardiovascular diseases, but also on chronic diseases for long-term health monitoring and patient management.

## 3. Methods

According to Xiao and Watson [34], literature reviews are essential for academic research. MacMillan et al. [35] stated that a systematic review provides a broad overview of a particular research topic. For this reason, literature reviews should have clearly defined inclusion and exclusion criteria. They should also present a comprehensive search that identifies all of the relevant literature, uses explicit and reproducible selection criteria for included studies, rigorously assess potential bias in the included studies, and systematically summarize the results of the included studies [35]. The purpose of a systematic review should be to answer an important, answerable question or to identify areas of high importance [36]. This review aims to map the landscape of IoT/IoMT technologies and machine learning techniques used for the detection, prediction, and monitoring of CVD.

This systematic review was guided by three research questions based on detecting, predicting, or monitoring CVD, as presented in Table 1. These research questions are based on the main contributions previously described in Section 1: IoT/IoMT devices for monitoring and predicting CVD; different types of CVD in the population; and machine learning applications for detecting, predicting, or monitoring CVD.

This review also follows the PICO(C) (population, intervention, comparison, and outcome) template to develop answerable, researchable questions by considering the elements listed in Table 2, always in accordance with the three research questions defined in Table 1.

### 3.1. Data Sources

For accessibility, we focused on reviewed articles from databases such as PubMed, IEEE Library, Springer Link, and Science Direct, in order to retrieve relevant JCR proposals using IoT or IoMT for CVD detection, prognosis, or surveillance. Our time-frame was between 1 January 2016 and 9 May 2023, in order to include technologies that are new and have not yet been discontinued. The search terms consisted of the keywords listed in Table 3, derived from the template elements defined in Table 2.

With the keywords described above, we proceed to construct the generic search string, which included some words to be searched only in the abstract (ab): (ab “cardiovascular diseases” or ab “heart disease” or ab “cardiovascular events” or ab “heart illness” or ab “heart condition”) and (“IoT” or “IoMT” or “machine learning” or “deep learning” or “data mining”) and (ab “early detection” or ab “detect” or ab “predict” or ab “monitor”) and (“wearable devices” or “devices”).

After retrieving the potential papers from the databases, we implemented the following inclusion criteria: (i) JCR studies only, (ii) articles written in English, and (iii) studies published between January 2016 and May 2023, based on compatibility and technological evolution. On the other hand, we applied the following exclusion criteria to the resulting set of papers: (i) Publication types other than JCR journal articles (posters, conferences, proceedings), (ii) articles whose full text was not in English, (iii) studies published before January 2016 and after May 2023, and (iv) articles detecting or predicting CVD by heart sounds. Due to accessibility issues, 24 articles were excluded from the PubMed, IEEE, Springer Link, and Science Direct databases, as they could not be downloaded.

### 3.2. Study Selection

**Figure 2:**
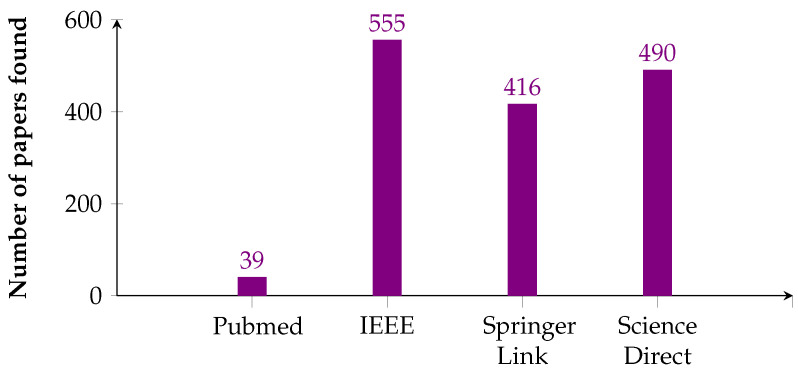
Papers found in digital libraries.

We obtained 1500 articles in total after applying the inclusion/exclusion criteria, of which 39 were from PubMed, 555 from IEEE, 416 from Springer Link, and 490 from Science Direct. Figure 2 shows the number of studies found in each digital library consulted.

**Figure 3:**
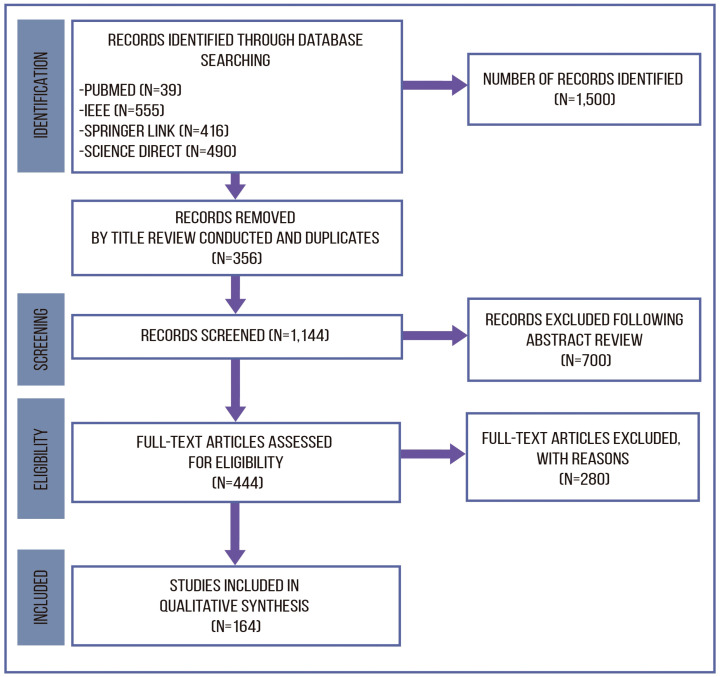
PRISMA flow diagram explaining the article selection process.

Studies were selected for this review using the four-step process (identification, screening, eligibility, and inclusion) according to the PRISMA flowchart shown in Figure 3. In the first stage of identification, we collected 1500 articles, of which 340 were reduced by title. Duplicates were removed at this stage, resulting in 1144 articles. In the screening stage, articles were thoroughly screened by reading the abstract to determine whether the article was focused on detecting, predicting, or monitoring CVD, and 700 papers that did not meet the inclusion criteria were excluded. At the eligibility stage, we read the full text of 444 papers to determine whether they were eligible for inclusion in the systematic review.

As shown in Figure 3, articles were excluded for several reasons (exclusion criteria) at the screening and eligibility stages. Each article was analyzed and classified by the authors, according to the following criteria: (i) area of study (monitoring, prediction, detection), (ii) disease (stroke, arrhythmia, atrial fibrillation, hypertension), (iii) data set used (public or private), (iv) approach (machine learning techniques), and (v) results (evaluation metrics: accuracy, precision, F1 score).

**Figure 4:**
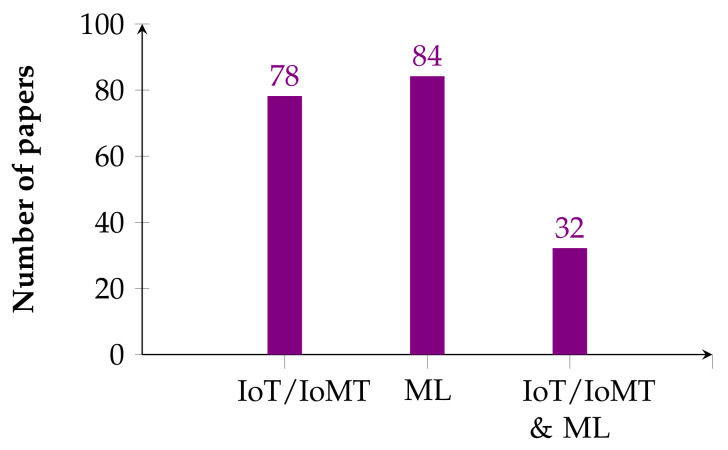
Histogram of the papers, divided into two main categories.

We conducted a peer review of the 162 potential studies answering the above three research questions to identify those that addressed the detection or prediction of cardiovascular disease and met the inclusion criteria. Appendix A shows these results, sorted by date. The 162 articles were divided into two main categories: CVD detection using IoT/IoMT and CVD detection using machine learning with public/private data sets. For the first category, we retrieved 78 papers, and 84 were retrieved for the second category. Figure 4 shows the JCR papers selected for this systematic review, divided into the two categories described above (i.e., papers that used IoT/IoMT technologies and machine learning-based CVD detection with public/private data sets). However, 32 of the papers in these two categories considered both IoT/IoMT technologies and machine learning-based CVD detection with public/private data sets.

### 3.3. Bibliometric Analysis

**Figure 5:**
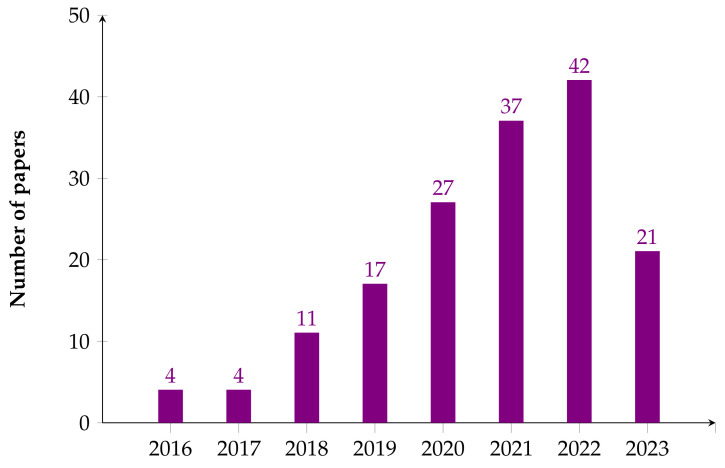
Histogram of the number of published papers per year.

The 162 papers included in the final analysis were published between January 2016 and May 2023 (Figure 5). We observed an increase in research output starting around 2019, with an even stronger increase in the following years. In 2022, we retrieved more papers related to the diagnosis, detection, prediction, and monitoring of CVD.

**Figure 6:**
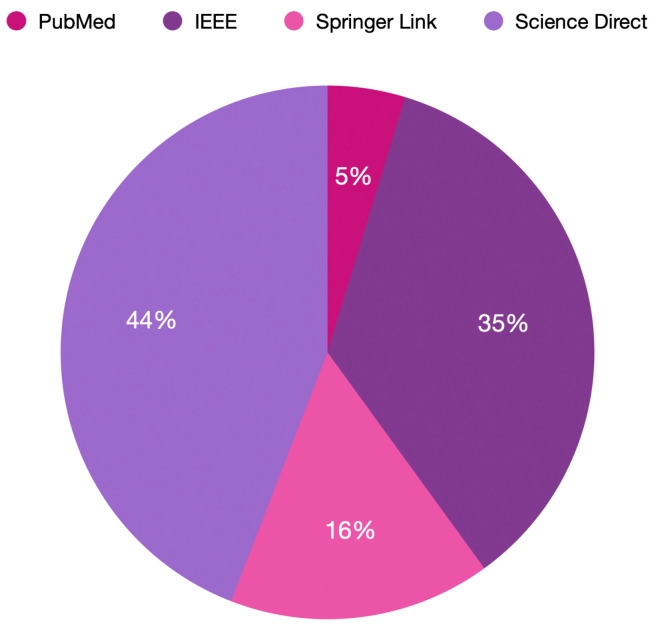
Percentage of articles selected by database.

The percentages of articles retrieved from the various databases are depicted in Figure 6. Science Direct had the major publication percentage (with 44%), followed by IEEE (with 35%). Springer Link had a 16% publication percentage, while PubMed contributed only 5% of the 164 papers selected for this review.

**Figure 7:**
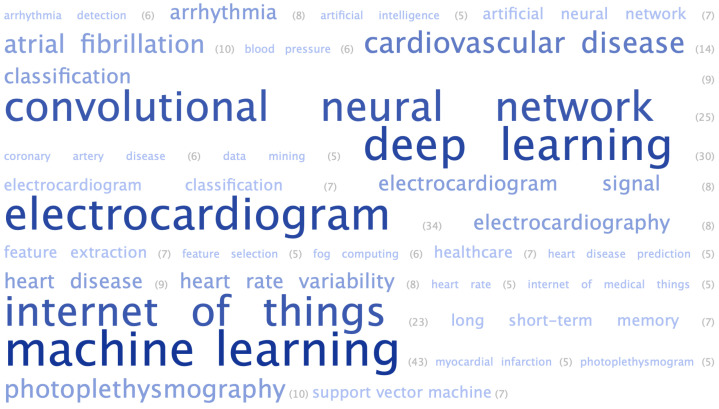
Word cloud for the most frequently used keywords.

We conducted a bibliometric analysis regarding authors, keywords, and journals. For each article, we obtained the database, journal, title, keywords or index terms, authors, number of citations, number of pages, and publication year. A word cloud was generated, according to the frequencies of keywords. The most frequently used keywords are highlighted in larger and bolder fonts, while the less frequently used keywords are highlighted in a smaller font in Figure 7. The keywords were grouped by similar words, and the most frequently used keyword was machine learning (43), followed by electrocardiogram (34), deep learning (30), and convolutional neural network (25).

**Figure 8:**
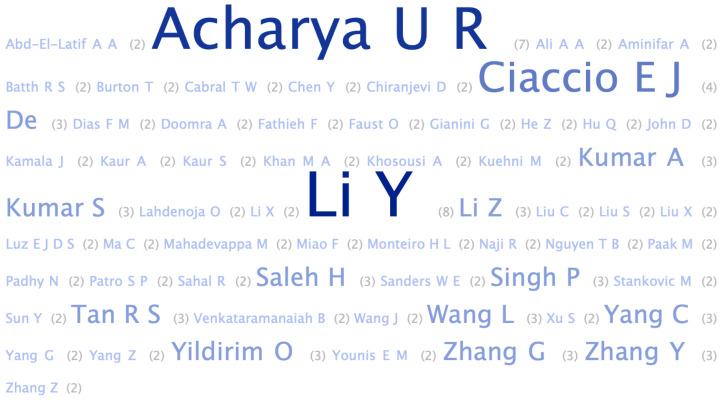
Word cloud for the most frequently published authors.

Figure 8 depicts the authors who most frequently published articles from the 164 selected papers. without removing duplicates, there were 797 authors. The most frequent authors were: Li, Y. (8), followed by Acharya, U. R. (7), and Ciaccio, E. I. (4). However, some authors seemed to have the same surname (e.g., Li) and a similar first name beginning with Y, such as Li, Ye (3); Li, Ya; Li, Yuanlu; Li, Yaowei (2); and Li, Yixuan. Acharya, U.R. was the only author with seven written articles related to CVD diseases. Similarly, Ciaccio, Edward J., have written four articles related to CVD disease. In total, Acharya, U.R. had 1074 citations in the seven papers, while Cicaccio, Edward J. had 433. The top 10 most-cited articles of the 164 articles that were selected are shown in Table 4.

**Figure 9:**
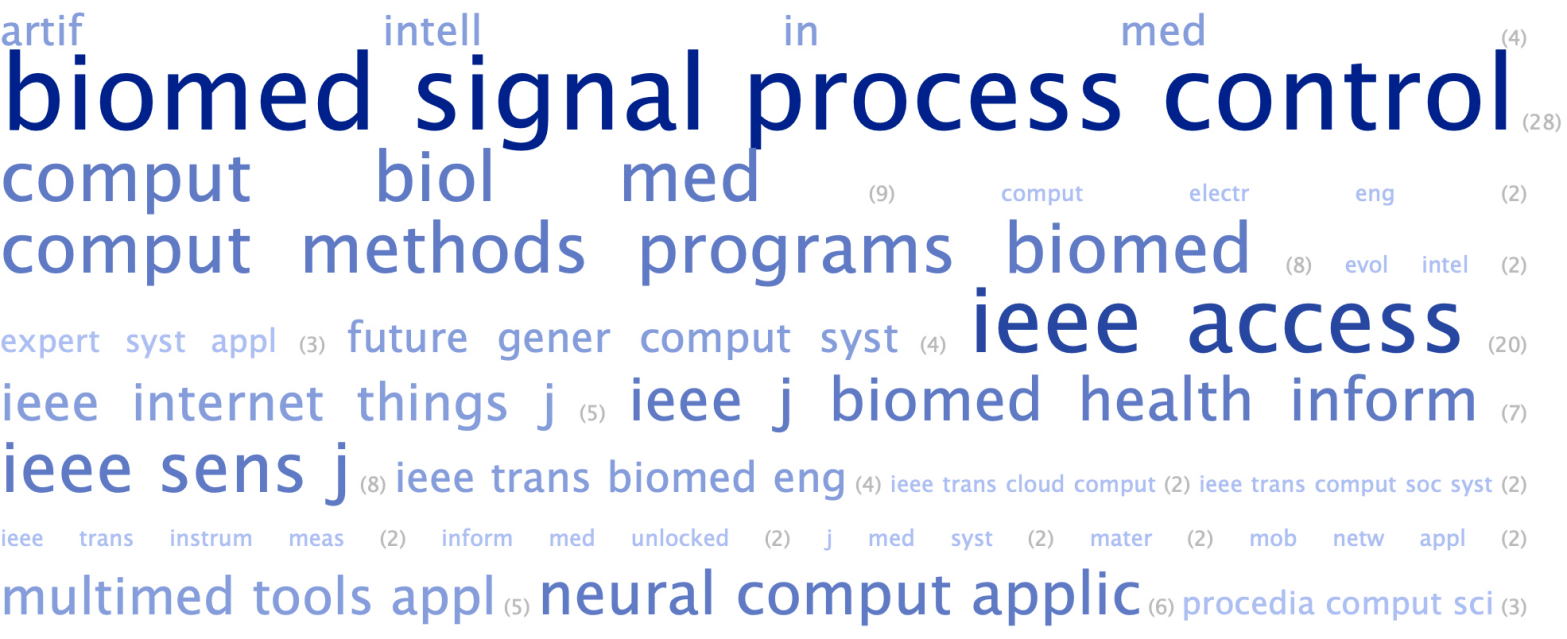
Word cloud for the most frequently published journals.

The most-cited articles were from the year 2019 and the IEEE database: Mohan, S., Thirumalai, G. and Srivastava, G. Science Direct had seven of the ten most-cited CVD-related articles: three from 2018, two from 2019, and two from 2020. The most-cited article had 888 citations, whereas the second and third had 579 and 432, respectively. The journal word cloud is shown in Figure 9.

The journal with the most articles related to CVD was *Biomed. Signal Process. Control* (28) from the Science Direct database. In second place was *IEEE Access* (20), followed by *Comput. Biol. Med.* (9) from the Science Direct database and *IEEE Sens. J.* (8).

## 4. Research on CVD Detection Using IoT/IoMT

The JCR papers selected as relevant for this review were classified according to the disease under study: Abnormality detection, arrhythmia, atrial fibrillation, blood pressure and hypertension, cardiovascular disease and heart disease, and another type of disease (e.g., aortic stenosis, arterial stiffness, chronic disease, chronic heart failure, myocardial infarction, and ischemic heart disease). Proposals may not address more than one disease, data set, or IoT/IoMT technology.

**Figure 10:**
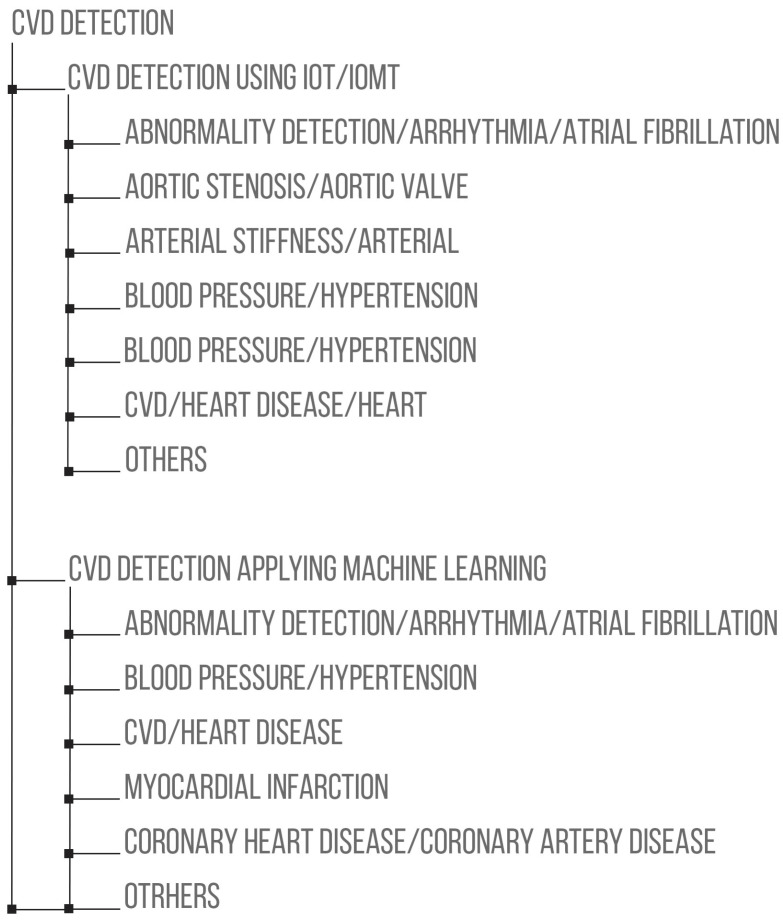
Organizational structure for the research on CVD.

We provide a summary of the wearable devices/smart devices/medical devices used, the machine learning techniques applied, and the results in terms of evaluation metrics. Figure 10 shows a pictorial representation of the organizational structure by disease for the research on CVD detection using IoT/IoMT and/or machine learning. Table 5 shows a summary of the disease classification. This classification led to the discovery that there is a wide variety of works using IoT/IoMT to promote the detection, prediction, or monitoring of CVD. The most commonly detected condition was CVD or heart disease in general (22.91%), arrhythmia (19.75%), other diseases (chronic heart failure, coronary artery disease, stroke, carotid artery disease; 8.69%), and blood pressure or hypertension (6.32%). On the other hand, aortic stenosis and arterial stiffness were the least-detected CVD conditions using IoT/IoMT.

### 4.1. Abnormality Detection and Arrhythmia

An abnormality or arrhythmia refers to an abnormal heartbeat rhythm; this means that the heart may beat too fast, too slow, or with an irregular rhythm. Arrhythmia is caused by changes in heart tissue, activity, and the electrical signals that control the heartbeat, which may be caused by damage from disease, injury, or genetics. Although there are usually no symptoms, some people experience an irregular heartbeat. Symptoms may include disorientation, difficulty breathing, fainting, or dizziness. The most common test used to diagnose arrhythmia is an electrocardiogram (EKG or ECG) [122]. Atrial fibrillation is the most common kind of treated heart arrhythmia. In atrial fibrillation, the atria—the upper, smaller chambers of the heart—do not generate normal electrical impulses and, therefore, do not contract. This causes the ventricles—the main pumping chambers of the heart—to beat rapidly and irregularly. Although atrial fibrillation is the most commonly sustained arrhythmia, it is not common. For example, in a 22-year study of 5191 adult men and women, only 2% developed chronic atrial fibrillation [123].

Table 6 shows the proposals classified by abnormality and arrhythmia detection, as well as atrial fibrillation. There were 26 proposals, but only Sannino and De Pietro [40] used a medical device (unspecified Holter) for condition monitoring. Keyanfar et al. [52] used a cardioverter–defibrillator device, Raheja and Manocha [60] used an ECG machine without specifying the model, and Fayyazifar et al. [62] used a MAC 550HD and a MUSE V9 (GE Healthcare, Chicago, IL, USA). Venkataramanaiah and Meenakshi [51] used biomedical sensors for the detection of arrhythmia without specifying the device. Two proposals used a smartphone (specifically, the Sony Xperia Z series model) (Sony, Tokyo, Japan): Mehrang et al. [70] and Lahdenoja et al. [66]. Mehrang et al. [70] also used a continuous five-lead telemetry ECG (Philips IntelliVue MX40). Cai et al. [68] and Hill et al. [69] used portable devices (KardiaMobile and a Mason linear ECG lead system, respectively) (AliveCor Inc., CA, USA), (CardioCloud Medical Technology, Beijing, China). Yang et al. [67] used an integrated analog front-end for heart rate monitoring, while Rawal, Prajapati, and Darji [71] used the device ZYNQ Ultrascale ZCU106 FPGA (Advanced Micro Devices, Inc., Santa Clara, CA, USA).

On the other hand, nine proposals used a microcontroller board, such as an Arduino Uno (Arduino, Scarmagno, Italy) and/or Raspberry Pi (Raspberry Pi Foundation, Cambridge, UK): Moghadas, Rezazadeh and Farahbakhsh [50], Al et al. [53], Farag [54], Scrugli et al. [55], Cheikhrouhou et al. [56], Sanamdikar, Hamde, and Atsutkar [57], Belaid et al. [59], Medhi, Ahmed and Hussain [61], and Misra et al. [64]. Zhao et al. [49] used a Lenovo smart ECG chest (Lenovo Group Ltd., Beijing, China), while Farahani et al. [48] used an unspecified set of electrodes and Yasin et al. [47] used a custom-made VA processor/SoC. Finally, Kumar et al. [58], Karthiga, Santhi, and Sountharrajan [63] and Shafi et al. [65] used an unspecified set of sensors nodes.

The best accuracy achieved was 99.94% by Al et al. [53] with AD8232 EKG Sensor (SparkFun Electronics, Niwot, CO, USA), Arduido board (Arduino, Scarmagno, Italy), and a Jetson Nano microcomputer (Nvidia Corporate, Santa Clara, CA, USA) using the R location correction (RLC) algorithm. The lowest accuracy was achieved by Yang et al. [67], with an integrated analog front-end using a linear kernel SVM. Other proposals have used metrics such as recall, precision, specificity, sensitivity, AUC, true positive rate, true negative rate, positive predictive, negative predictive, and ROC.

### 4.2. Aortic Stenosis

Aortic stenosis is an obstruction of blood flow from the left ventricular outflow tract which can occur at various levels, including at the aortic valve (valvular aortic stenosis), above (supravalvular aortic stenosis), or below the semilunar valve (subvalvular aortic stenosis). However, the clinical presentation of shortness of breath, syncope, and/or chest pain may be identical. Patients may have a systolic ejection murmur that is constant or changes with certain maneuvers (as in hypertrophic obstructive cardiomyopathy), as well as a variable intensity of the second heart sound, depending on the severity of the obstruction [125].

In Table 7, we list the proposals classified by aortic stenosis detection using IoT/IoMT. Yang et al. [72] used a three-axis accelerometer and three-axis gyroscope employing three classifiers: Decision tree, random forest, and neural network. Petrou et al. [73] used a non-implantable mixed-flow turbodynamic blood pump (Deltastream DP2, Xenios AG, Helibronn, Germany) with a cardiac output estimation pipeline, while Cheng et al. [74] used an ultrasound with two 3D convolutional neural networks (GE Vingmed Ultrasound AS, Norway Health Tech, Horten, Norway). The best performance was obtained by Yang et al. [72] combining seismo-cardiography (SCG) and gyro-cardiography (GCG) features with a random forest classifier (98.96%). The lowest accuracy was achieved by Cheng et al. [74], with an accuracy of 83%.

### 4.3. Arterial Stiffness

Arterial stiffness is associated with changes in the structure and function of the arteries. Increased arterial stiffness is caused by impaired smooth muscle action, resulting in altered arterial dilation and constriction and increased blood pressure. In older adults, it is associated with isolated systolic hypertension and greater CVD risk. In addition to the functional regulation of blood pressure, structural changes within the vessels contribute to arterial stiffening. These changes include thickening and re-modeling within each of the three layers of the artery [126].

Table 8 shows the proposals classified by arterial stiffness detection using IoT/IoMT. Miao et al. [75] used a medical device OMRON BP-203RPE III (OMRON Industrial Automation, Kyoto, Japan) and multi-variate linear regression to achieve an accuracy of 89% for vascular age. A back-propagation neural network was employed to achieve an accuracy of 94% for CVD risk estimation. Dami and Yahaghizadeh [76] used a different set of unspecified sensors to achieve an accuracy of 88.42% through a combination of principal component analysis, deep belief networks, and a long short-term memory model. Asorey et al. [77] proposed a diagnostic system with an unspecified bio-sensors that can issue an alert within three hours if an artery appears to be blocked, and release medication in the next three hours if the artery is really blocked. Only two proposals have reported evaluation metrics such as accuracy. The lowest reported accuracy was 88.42% by Dami and Yahaghizadeh [76], while Miao et al. [75] reported 89% and 94%.

### 4.4. Blood Pressure and Hypertension

Hypertension—or high systemic arterial blood pressure—is a common disease that develops when blood flows through the arteries at higher than normal pressure. There are two numbers that describe blood pressure: systolic and diastolic. The pressure created when the ventricles pump blood out of the heart is called systolic pressure, while the pressure between heartbeats—when the heart is filled with blood—is called diastolic pressure. Blood pressure changes throughout the day, based on activities [127]. The 2003 Guidelines of the European Society of Hypertension/European Society of Cardiology consider a patient as hypertensive when either the systolic blood pressure or diastolic blood pressure value is ≥140/90 mmHg [128].

Table 9 provides a summary of the proposals for blood pressure and hypertension detection using IoT/IoMT. Miao et al. [85] used a vital signs monitor (Benevision N12 Mindray), while Lan et al. [81] used a ring PPG, an accelerometer, and a Zigbee device on a custom-built device. Yan et al. [84] used a blood pressure monitor Finometer MIDI Model II, (Finapres Medical Systems B.V., Amsterdam, The Netherlands) and a pulse oximeter (Contec Inc., Qinhuangdao, China). On the other hand, Mohebbian et al. [82] and Riaz et al. [83] used the Raspberry Pi (Raspberry Pi Foundation, Cambridge, UK) and Arduino (Arduino, Scarmagno, Italy) microcontroller boards. Forkan et al. [78] used an accelerometer, a GPS, an ECG, and a blood pressure monitor without specifying the devices. Zhang et al. [80] used three electronic components from TX Instruments (ADS1299EEG-FE, AFE4490SPO2, MSP430F5529IPN, TX Instruments, Dallas, TX, USA). Finally, Ghosh et al. [79] used a custom-built device based on the principle of impedance plethysmography with an auto-adaptive algorithm based on impedance cardiography signals.

In terms of accuracy, the best performance was 99.78% for Patient 2 by Forkan et al. [78], achieved using a decision tree J48 as the classifier. The lowest performance was 81.52%, achieved using the radial basis function in the same proposal [78]. Other works have used the mean error, root mean square error, or mean absolute error to evaluate their results. For example, Zhang et al. [80] used mean error, mean absolute error, and root mean square error for heart rate estimation. The results were 0.8 ± 2.7 beats per minute for mean error and standard deviation, 1.8 beats per minute for mean absolute error, and 2.8 beats per minute for root mean square error. Conversely, Mohebbian et al. [82] achieved 3 ± 0.7 mmHg for systolic blood pressure by root mean square error, 2.2±0.7 mmHg for diastolic blood pressure by root mean square error, 4.4±1.0 mmHg for systolic blood pressure by mean absolute error, and 2.9±1.2 mmHg for diastolic blood pressure by mean absolute error. Yan et al. [84] used the mean absolute error for systolic and diastolic blood pressure, obtaining 0.043 mmHg and 0.011 mmHg, respectively. The mean blood pressure obtained was 0.008 mmHg. Miao et al. [85] used the mean difference ± standard deviation accuracy, which was obtained as −0.22±5.82 mmHg for systolic blood pressure. The mean arterial pressure mean difference ± standard deviation accuracy obtained was −0.57±4.39 mmHg. Finally, the mean difference ± standard deviation accuracy obtained was −0.75±5.62 mmHg for diastolic blood pressure. Ghosh et al. [79] predicted systolic BP, diastolic BP, and heart rate accuracies of ±2.33 mmHg, ±3.60 mmHg, and ±2.88 mmHg beats per min, respectively.

### 4.5. Cardiovascular Disease and Heart Disease

Some proposals have not specified the disease or disorder to be detected, instead considering cardiovascular disease or heart disease as a general disease. In Table 10, we list the proposals that correspond to this classification using IoT/IoMT.

There were 31 proposals corresponding to this classification. The majority used sensors or microcontrollers such as Raspberry Pi or Arduino in combination with heart rate sensors, temperature sensors, blood pressure sensors, SpO${}_{2}$ sensors, or even temperature sensors. Six proposals used medical devices such as those manufactured by OMRON, Holter, Zephyr, or even Polar. Five proposals used wearable devices, but did not provide further details. There were two proposals which used a radar and a chip in combination with a mixed-signal neural network: Reservoir-computation (RC-NN) and long short-term memory (LSTM), respectively. A total of 20 proposals used accuracy as an evaluation metric, 8 of them used specificity, 4 used sensitivity, 10 used precision, 2 did not specify the evaluation metric used, while others reported median error, root mean-squared difference, the worst performance with SI score, train loss/valid loss, and 1 reported 90% less energy consumption. Clifford et al. [97] proposed an application cloud infrastructure to provide detailed information on the physical activity, behaviors, and psycho-social and physiological status of urban African American young adults without providing further details.

The best accuracy achieved was 99.58% by Demirel, Bayoumy, and Faruque [110]. Conversely, the lowest accuracy was 60.98%, obtained by Moradkhani, Broumandnia, and Mirabedini [105] using a probabilistic neural network. In terms of sensitivity and specificity, the best performance was that of Demirel, Bayoumy, and Faruque [110], who used a convolutional neural network and obtained 99.2% and 99.4%, respectively. The lowest performance in terms of sensitivity was 45.5%, obtained by Boursalie, Samavi, and Doyle [88] using a support vector machine classifier. The lowest specificity was 55.1%, again obtained by Moradkhani, Broumandnia, and Mirabedini [105].

### 4.6. Others

In this subsection, we group the proposals that focused on other diseases of lesser frequency (i.e., chronic heart failure, myocardial infarction, coronary artery disease, carotid artery, saturated oxygen, stroke disease, ECG abnormalities, and ECG noise). In Table 11, we list the proposals that correspond to different diseases using IoT/IoMT.

Two proposals corresponded to chronic heart failure: those of Aranki et al. [111] and Hanumantharaju et al. [112]. Meanwhile, Sopic et al. [113] and Tozlu et al. [114] focused on myocardial infarction detection. Verma et al. [115] detected coronary artery disease. The study of Yu et al. [118] was the only proposal to classify stroke, while only Rodriguez et al. [117] classified saturated oxygen and Sahani et al. [119] focused on carotid disease. Ying et al. [116], Sivapalan et al. [121], and Rahman et al. [120] focused on ECG abnormalities and ECG noise.

The best performance in accuracy (99.8%) was achieved by Yu et al. [118], using a deep neural network. The worst was 97.74%, achieved by Aranki et al. [111] in combination with an SVM classifier. The rest of the proposals reported precision, sensitivity, specificity, recall, F1-score, and root mean square in combination with classifiers such as SVM, random forest, long short-term memory, deep neural network, artificial neural network, an R-peak detection algorithm, and ResNet-9 semi-supervised learning. Sahani et al. [119] reported a reduction of the data dropout rate (21.09%) and an increment in the number of R-peak detections (15.33%).

The devices used to detect CVD diseases included sensors (e.g., ECG, EEG, EMG, humidity sensor, electrochemical gas sensor, temperature sensor, motion sensor) and Raspberry Pi (Raspberry Pi Foundation, Cambridge, UK), ARM M4F (Nordic semiconductor, Trondheim, Norway), 12C master (Custom-built device) microcontrollers. Hanumantharaju et al. [112] used a different set of unspecified sensors, and Rodriguez et al. [117] used a custom-built signal acquisition device.

## 5. Examples of CVD Detection Utilizing Machine Learning

This section lists the articles that used machine learning exclusively to detect, predict, or monitor CVD. The papers were classified in terms of the following: abnormality detection, arrhythmia or atrial fibrillation, blood pressure or hypertension, cardiovascular disease, heart disease or heart failure, myocardial infarction, coronary heart disease or coronary artery disease, and other kinds of disease related to CVD. We provide a summary of the data sets used, the machine learning techniques applied, and the results obtained according to the evaluation metrics (accuracy, sensitivity, specificity, F1-score). Table 12 shows a summary of the disease classification, which led to the determination of what type of machine learning techniques have been used to detect, predict, or monitor CVD.

The most commonly detected conditions were arrhythmia (38.82%) and cardiovascular disease (29.41%). Conversely, the fewest were cardiomyopathy (including hypertrophic cardiomyopathy), ischemic heart disease, valvular heart disease, left ventricular hypertrophy, chronic heart failure, and stroke, classified in the 8.23% of other diseases.

### 5.1. Abnormality Detection and Arrhythmia

Table 13 summarizes the abnormality and arrhythmia detection proposals using only machine learning approaches. There were 33 proposals, 26 of which used the MIT-BIH or PhysioNet data sets. Other data sets used were privately provided by hospitals or health centers. On the other hand, the machine learning approaches that were most frequently used were neural networks such as convolutional neural networks, long short-term memory, deep neural networks, bidirectional long short-term memory, lead convolutional neural networks, deep residual neural networks, densely connected convolutional networks, multi-scale fusion convolutional neural networks, and one-dimensional neural networks. Other techniques included SVM, *k*NN, random forest, genetic algorithms, bacterial-foraging optimization, and particle swarm optimization.

In terms of evaluation metrics, some of the higher values reported were achieved with neural networks. Haleem et al. [142], for example, achieved 100% accuracy for congestive heart failure events and sudden cardiac deaths. The rest of the proposals reported accuracy in the range of 98–99%. Other metrics reported included F1-score, precision, recall/sensitivity, specificity, AUC, positive productivity, and positive predictive value. Focusing on sensitivity and specificity values, the best performance for the former was 99.8%, obtained by Ma et al. [155], while the best specificity was 99.7%, obtained by Tung et al. [149]; both proposals used convolutional neural networks.

In general, the results ranged between 81% and 99%. However, Dias et al. [141] and Li, Qian, and Li [151] reported lower precision values: 36.8% and 65.88%, respectively. Li, Qian, and Li [151] also reported a lower sensitivity (35.22%). Both proposals achieved lower results in these metrics for supraventricular segment or ectopic beat.

### 5.2. Blood Pressure and Hypertension

Table 14 shows a summary of the proposals for blood pressure and hypertension detection using only machine learning approaches. Ten proposals were analyzed in this category for detecting blood pressure or hypertension. Nine proposals used the MIT-BIH data set, while only one proposal used the UCI data set. The more frequently used machine learning techniques included recurrent neural networks, convolutional neural networks, bi-directional long short-term memory, long short-term memory, multi-scale fusion neural networks, deep learning, artificial neural networks, and decision trees. In terms of results, only three proposals included accuracy as a metric: Alkhodari et al. [158], Zhang et al. [163], and Kim et al. [167]. Alkhodari et al. [158] and Kim et al. [167] reported an accuracy above 91%, while Zhang et al. [80] reported an accuracy for systolic blood pressure in normal, atrial fibrillation, and coronary arteriosclerosis subjects. Other proposals focused on MAE, RMSE, and mean absolute error or standard deviation metrics. The majority reported results for systolic and diastolic blood pressure, but Alkhodari et al. [158]; Landry, Peterson, and Arami [159]; Saleh et al. [160]; and Mahmud et al. [166] reported only general results. In terms of accuracy, precision, F1-score, sensitivity, and specificity metrics, Alkhodari et al. [158] obtained a better accuracy (97.08%) than Kim et al. [167] (91.44% and 94.66% for systolic and diastolic BP); however, Kim et al. [167] achieved a better performance in terms of sensitivity and specificity (above 94% for specificity and above 70.17%for sensitivity).

### 5.3. Cardiovascular Disease/Heart Disease

In Table 15, we list the proposals that classified a common disease (cardiovascular disease or heart rate disease) using only machine learning approaches. There were 25 proposals, 14 of which used the PhysioNet and MIT-BIH data sets, 12 used the UCI data set, 5 used private data sets, and 2 used the Framingham data set. Many machine learning approaches were used to classify cardiovascular disease, including SVM, Naïve Bayes, random forest, multi-layer perceptron, hybrid random forest, XGBoost, convolutional neural network, long short-term memory, particle swarm optimization, neuro-fuzzy inference system, deep neural network, beetle swarm optimization, twin SVM, deep neural network, neural network, artificial neural network, decision tree, bidirectional long short-term memory, sine cosine *k*NN, gradient boost, deep learning, growing multi-layer network, and other approaches for big data processing, ensemble-based feature selection, and recursive feature elimination.

The most common metrics used for evaluation were accuracy, precision, recall, F1-score, sensitivity, specificity, AUC, MAE, and RMSE. The positive predictive value, the estimation error, and the equal rate of error was also used to evaluate the proposals. The best accuracy achieved was 99.45%, in combination with a modified salp swarm optimization–adaptative neuro fuzzy inference system proposed by Khan and Algarni [45]; they also achieved good precision (96.54%). Deepika and Balaji [182] used a multi-layer perceptron to obtain good performance in terms of accuracy, sensitivity, F1-score, specificity, and Kappa (all of them above 96.40%). The two proposals which reported lower performance were those of Theerthagiri [181] and Stankovic et al. [185], achieving a performance above 83% but lower than 89% in accuracy, precision, F1-score, and recall. Theerthagiri [181] also obtained lower performance in AUC, MSE, and RMSE, compared to other proposals.

### 5.4. Myocardial Infarction

A myocardial infarction (MI) is characterized by a typical rise and/or fall in cardiac troponin, with at least one value above the upper reference limit of the assay, and at least one other characteristic associated with ischemia. Also known as myocardial infarction, it occurs when the flow of oxygenated blood to a segment of the heart muscle is suddenly interrupted and the heart is deprived of oxygen. The segment of heart muscle begins to die if blood flow is not immediately restored [206].

Table 16 lists the six proposals that classified myocardial infarction using only machine learning approaches. Three of them used convolutional neural networks, one a long short-term memory, and two used traditional approaches: SVM and random forest. The most-used data sets were the Physikalisch-Technische Bundesanstal and PhysioNet data sets. Xiao et al. [192] used medical records retrieved from a hospital, but obtained lower performance in terms of accuracy and AUC (82% and 85%, respectively). The best performance was obtained by Fatimah et al. [193] in combination with *k*NN, where the accuracy, sensitivity, and specificity obtained were all above 99.94%. Meanwhile, Deng et al. [190] used a convolutional neural network and achieved good performance (above 98.1%), and Ibrahim; et al. [191] obtained a performance above 93.4% with an XGBoost classifier.

### 5.5. Coronary Artery Disease/Coronary Heart Disease

Coronary heart disease is a condition in which the heart muscle—or myocardium—does not receive enough oxygen as the coronary arteries do not have an adequate blood supply. Coronary artery obstruction occurs when the arteries become stiff and narrow due to the build-up of fatty deposits (plaques). These fatty deposits are mainly composed of cholesterol and fibrin and, when these deposits predominate, it is called atherosclerosis [207].

Table 17 summarized the proposals for coronary heart disease detection using only machine learning approaches. There were four proposals, each of which considered different approaches: logistic regression, elastic net, SVM, random forest, XGBoost, multi-layer perceptron, k-NN, bagging, binary logistic classification, Naïve Bayes, boosting, and random forest. The data sets also were from different sources, including Framingham, PhysioNet, and a cardiovascular disease data set. In terms of evaluation metrics, the most used were AUC, accuracy, sensitivity, and specificity. The best performance in terms of sensitivity was 100%, obtained by Dash et al. [198]. Meanwhile, Masih, Naz, and Ahuja [196] achieved better accuracy and specificity: 96.50% and 98.28%, respectively.

### 5.6. Others

In this section, diverse proposals from different diseases are grouped into the same table (Table 18), for a total of seven proposals. For example, Dai, Hwang, and Tseng [202] focused on classifying cardiomyopathy; Smole et al. [204] considered hypertrophic cardiomyopathy; Elias et al. [200] considered valvular heart disease; Duffy et al. [201] considered left ventricular hypertrophy; Loeffler and Starobin [203] considered ischemic heart disease; Li et al. [199] considered chronic heart disease; and Pathan et al. [205] considered stroke.

The most-used machine learning techniques were convolutional neural networks (1D, 3D, deep convolutional). Other proposals used more traditional techniques, such as random forest, SVM, and boosted trees. Elias et al. [200] used AUC-ROC as an evaluation metric, obtaining 88% for detecting aortic stenosis. Meanwhile, Duffy et al. [201] reported an AUC of 98% for hypertrophic cardiomyopathy. Dai, Hwang, and Tseng [202] achieved 99.84% accuracy—the highest of the seven proposals. Pathan et al. [205] used a support vector machine classifier and obtained the precision, sensitivity, F1 score, and accuracy of each attribute in the Mckinsey data set (i.e., gender, age, hypertension, heart disease, ever married, work type, residence, glucose level, BMI, and smoking status).

## 6. Results and Discussion

As a result of this systematic review, 162 articles were analyzed and classified into two categories: those using IoT/IoMT technologies and those applying machine learning techniques. We obtained 78 proposals for the first category and 84 for the second. In each of these categories, we evaluated the articles according to six parameters: Study area, disease, data set used, wearable device/smart device/medical device, approach, and outcomes. We utilized these categories to answer the research questions presented in Section 3.1. For RQ1, we collected information about wearable devices/smart devices/medical devices; for RQ2, we considered the data set used, approach, and results; and, finally, for RQ3, we focused on the predicted disease.

Our reading of the 162 papers revealed that several devices have been used to monitor or detect CVD, including smartphones, microcontroller boards with sensors, and even experimental devices built specifically for CVD. We also found that neural networks were one of the most commonly used machine learning approaches, including convolutional neural networks, long short-term memory, bidirectional long short-term memory, multi-layer perceptrons, and deep neural networks.

Unlike other works, our comprehensive review details the machine learning methods used in conjunction with wearable/smart devices/medical devices and data sets for the detection, prediction, and/or monitoring of CVD, including the most-researched disease types (i.e., abnormality detection/arrhythmia, aortic stenosis, arterial stiffness, atrial fibrillation, blood pressure/hypertension, heart disease in general, chronic heart failure, myocardial infarction, coronary heart disease and stroke), in a single work. We place focus on the technology used, presented in the form of wearable devices/smart devices/medical devices and machine learning approaches for the classification of CVD, rather than a specific disease. Our results indicate that CVD/heart disease in general was the disease most commonly captured by wearable devices/smart devices/medical devices. In contrast, abnormality detection/arrhythmia was the disease most focused on when applying machine learning methods, thanks to the public MIT-BIH data set. We found that there is a need for more public CVD data sets, as the MIT-BIH and Cleveland-UCI public data sets were the most widely used. On the other hand, we also found that neural networks were the most-used approach, while other approaches included traditional machine learning methods, statistical approaches, and optimization techniques.

We now summarize the main results presented in Section 4 and Section 5, with the aim of answering the research questions introduced in Section 3. The discussion is divided into three parts, related to the detection, prediction, or monitoring of CVD using IoT/IoMT technology or machine learning techniques.

### 6.1. Research Question 1

RQ1: What types of devices with IoT and IoMT technologies have been used to detect and predict cardiovascular disease using machine learning? was focused on discovering the technologies and wearable devices/smart devices/medical devices used to detect or monitor CVD in real-time as preventive care.

The technologies discovered in the systematic review included medical devices, smartphones, microcontroller boards, sensors, smartwatches, radar, and wearable devices. These technologies were combined with frameworks, applications, and systems to monitor or detect CVD. Some technologies used the cloud to store and process the data obtained from the devices. In these cases, data privacy and security are also important, considering the importance of patient confidentiality. The devices and the system need to be secure and reliable, as they manage medical and physiological data. Preferably, the devices must be FDA-approved to avoid significant measurement errors; however, in monitoring cases, some patients may not be able to afford medical devices and a framework including commercial wearable devices needs to be developed. This is especially important for rural communities which, in some cases, do not have easy access to health services. In this case, the systems must be responsive to end-user requests for data extraction, downloading, and consultation. They must also be non-intrusive and easy to wear (body placement).

After analyzing the 162 papers selected as relevant, we observed an increase in relevant research output starting around 2019. These proposals started to use IoT/IoMT devices more frequently for the monitoring of chronic diseases in real-time, with promising results obtained after the COVID pandemic. Some of the proposals included in the review used IoT/IoMT technologies in combination with clinical decision support systems. The use of these systems can help clinicians to assess the risk of heart disease and provide treatments to further manage risk. Implementing models into decision support systems can improve preventive care, allow for the collection and analysis of data in real-time, and reduce the likelihood of misdiagnosis. The proposed models uncovered in this systematic review were developed to provide high-performance predictions of the presence or absence of heart disease, given the current condition of the participants.

Other proposals used frameworks in combination with IoT/IoMT technologies. Khan [43] proposed modules in a prediction system that integrates hardware devices, microcontrollers, and LoRa communication hardware to transmit data to the cloud system. Forkan et al. [78] presented a generalized framework for personalized healthcare connected to a personal cloud server. The local server only collects low-level data (e.g., ECG data, blood pressure monitor data, accelerometer data) from the ambient assisted living system, then forwards them directly to the context aggregator or the personal cloud server.

Microcontrollers were one of the most common devices used for the detection or prediction of CVD. The two most common microcontrollers were Arduino and Raspberry Pi, in combination with various sensors (e.g., photopleytmography, ECG, heart rate, temperature, electroencephalagram, SpO${}_{2}$, respiration, EMG). Some authors have also developed healthcare monitoring systems presenting good results in the evaluation metrics. In such instances, the medical devices performed well in the evaluation process, such as Holter devices, biomedical sensors, and Omron, achieving an accuracy of over 94%. To the contrary, sensors and microcontroller boards only achieved an accuracy above 72%. Other devices, such as the Lenovo Smart ECG Vest, achieved an accuracy above 86%. In terms of specificity, the Kardiamobile and SmartCardia devices achieved 74.9% and 78.82%, respectively. The Zigbee device achieved an accuracy of 92.11%, while the SmartCardia NYU device achieved accuracy, precision, specificity, F1-score, and sensitivity values above 90%.

The most convenient device we detected for the monitoring of CVD were smartphones, being a non-intrusive device that most of people have access to. Unfortunately, these devices do not provide good measurements, when compared to medical devices, and wrong measurements can lead to mortally serious outcomes. Ideally, only medical-grade devices should be used to monitor chronic diseases such as CVD, precisely due to the importance of acting in a timely manner in the event of an emergency. People who monitor their health on a daily basis can survive if the system alerts them (or their caregivers) to an impending stroke or heart attack. Devices that use sensors or microcontrollers provide a good solution for an unobtrusive device, but a huge amount of testing is necessary to approve the reliability of their measurements. The goal of any device that monitors a chronic disease is to prevent an incoming emergency and send alerts to a caregiver, relatives, and even hospitals, in order to prevent a loss of life.

### 6.2. Research Question 2

RQ2: What machine learning techniques have been applied to detect and predict cardiovascular disease? was focused on determining the machine learning approaches used to classify, predict, and/or analyze CVD data, in order to compare the approaches as well as the evaluation metrics and their results.

Machine learning techniques allow for the exploration of an immense amount of data, feeding a computer algorithm to analyze the input data. As such, they allow software applications to predict diseases. As described in Section 5, many machine learning techniques have been used to detect CVD. Some of the most popular approaches were neural networks and classifiers such as random forest, XGBoost, *k* nearest neighbors, or support vector machine. Other proposals have used a combination of techniques, such as a modified salp swarm optimization–adaptive neuro-fuzzy inference system (MSSO-ANFIS) and a beetle swarm optimization–adaptive neuro-fuzzy inference system (BSO-ANFIS). It was found that the proposals using a combination of machine learning approaches generally obtained good performance in the accuracy metric, over 96%. Meanwhile, those utilizing neural networks (deep neural networks, multi-layer perceptrons, convolutional networks, long short-term memory) obtained accuracy values above 90%. Some recent algorithms have been applied to CVD detection; for example, particle swarm optimization has been used in two proposals—those of Dang et al. [133]—and obtained an accuracy of above 95%.

Other approaches, such as linear-kernel SVM and hybrid random forest with a linear model, have been used for CVD detection and achieved good performance in terms of evaluation metrics. Linear kernel SVM achieved specificity and sensitivity of 99.6% and 79.3%, respectively, while the hybrid random forest achieved an accuracy of 88.7%. The proposals that used an SVM as a machine learning classifier obtained an accuracy above 88%.

We noticed that the best performance in evaluation metrics was obtained with a combination of techniques and neural networks. The most popular classifiers (Random Forest, SVM, XGBoost) achieved good performance. Some of the neural networks used included deep neural networks, convolutional neural networks, long short-term memory, multi-scale fusion convolutional neural networks, end-to-end deep multi-scale fusion convolutional neural networks, lead convolutional neural networks, recurrent neural networks, bidirectional long short-term memory, deep learning-modified neural networks, and convolutional neural network–long short-term memory. The most widely used neural networks were the convolutional neural network and long short-term memory.

In general, deep learning using neural networks, as some of the most-used techniques in the state-of-the-art, obtained good results. However, some of the proposals solely focused on the accuracy metric, describing how the model performs across all classes without focusing on other metrics such as sensitivity, specificity, precision, or F1-score. Some algorithms based on the behavior of animals were used to detect or predict CVD, such as the dragonfly algorithm used by Deepika and Bajaji [182], which obtained 97.47% accuracy, 98.92% sensitivity, 96.45% F1-score, 96.47% specificity, and 96.75% kappa. Another example is the study of Singh et al. [172] who obtained 99.91% accuracy, 99.37% precision, 99.4% specificity, and 99.21% sensitivity for heart disease classification using a beetle swarm optimization–adaptive neuro-fuzzy inference system. Raj [171] also used an animal-inspired algorithm in the modified salp swarm optimization–adaptative neuro-fuzzy inference system to obtain 99.45% accuracy and 96.54% precision. Khan and Algarni [45] used the biologically inspired particle swarm optimization algorithm to achieve 99% accuracy. Similarly, Kora, Abraham, and Meenakshi [132] used the particle swarm optimization in addition to a bacterial-foraging optimization to obtain 99.1% accuracy for atrial fibrillation classification applying wavelet transform, 98.9% accuracy for myocardial infarction using SVM classifier, and 99.3% accuracy for bundle branch block using the same classifier.

### 6.3. Research Question 3

RQ3: What types of diseases have been detected and predicted? was focused on gathering the cardiovascular diseases that are more reliable to monitor or detect through the use of devices/wearable devices/smart devices/medical devices. Likewise, we also aimed to determine the diseases that are hardly monitored using IoT/IoMT technologies, as an opportunity to develop real-time systems or applications.

The most frequently detected diseases using IoT/IoMT technologies were cardiovascular diseases in general, arrhythmia, and blood pressure/hypertension. On the other hand, the most common conditions detected by machine learning alone were arrhythmia, cardiovascular disease in general, and blood pressure. A total of 59 proposals focused on CVD in terms of arrhythmia, 55 on CVD, 18 on blood pressure/hypertension, 8 on myocardial infarction, 6 on coronary artery disease, 3 on chronic heart disease, 3 on aortic stenosis, 3 on arterial disease, 2 on stroke, 2 on cardiomyopathy, 1 on valvular heart disease, 1 on left ventricular hypertrophy, 1 on ischemic heart disease, 1 on saturated oxygen, and 1 on carotid disease.

We noticed that some proposals did not specify the detected disease and considered cardiovascular disease as a general disease, which was one of the most-detected diseases using IoT/IoMT technologies and using only machine learning. However, these proposals utilized public data sets, such as UCI Cleveland and PhysioNet MIT-BIH. The less common diseases detected included chronic heart disease, myocardial infarction, coronary artery disease, stroke, carotid disease, ischemic heart disease, left ventricular hypertrophy, cardiomyopathy, and valvular heart disease.

A significant number of proposals found, detected, and/or predicted CVD, arrhythmia, and hypertension, but there is an opportunity to detect specific diseases, such as stroke or myocardial infarction, that may be useful to predict in the context of healthcare systems.

Other observations from this systematic review are as follows. Most of the data sets used were from the UCI and PhysioNet sites, while data sets from hospitals and private companies were included in some cases. The devices used to monitor or diagnose CVD ranged from medical devices to smart watches and microcontroller cards. Finally, some devices were developed for specific diseases and evaluated in specific groups (including control groups).

### 6.4. Future Trends

Over the past few years, several trends in Internet of Things (IoT) and Internet of Medical Things (IoMT) technologies for CVD classification and prediction have emerged. These trends include clinical decision support systems, wearable devices, data analytics, data security, remote patient monitoring, and risk assessment combined with predictive analytics.

A clinical decision support system is a computer-based tool designed to assist healthcare providers in making clinical decisions by providing relevant information, knowledge, and recommendations at the point of care. One of their benefits is providing healthcare providers with real-time insights and recommendations for the diagnosis and management of CVD. These systems require the development of intelligent algorithms and decision support tools to help healthcare professionals make accurate and timely decisions. They also integrate patient-specific data from multiple sources, such as electronic health records (EHRs), medical imaging systems, laboratory results, and IoT/IoMT devices. Such integration enables the system to easily provide a comprehensive view of a patient’s health information at any time and provide treatment recommendations based on established guidelines and individual patient characteristics. It can suggest potential diagnoses, differential diagnoses, help to rule out certain conditions, and provide appropriate medications, dosages, treatment plans, and lifestyle changes for the management of CVD by analyzing patient data and symptoms. The key benefit of such a system is that they can generate alerts and reminders for healthcare providers, notifying them of critical information such as abnormal test results, drug interactions, or upcoming preventive screenings. These reminders help to ensure timely intervention and adherence to clinical guidelines. It can also help to monitor patient progress and provide follow-up recommendations. Remote patient monitoring can track vital signs, lab results, and IoT/IoMT data to assess treatment effectiveness and suggest adjustments as needed. However, the wearable devices, smart devices, or sensors integrated into microcontroller boards must be reliable and highly accurate—and preferably FDA-approved—as the vital signs of patients can change rapidly in the context of chronic disease, and a major change may even lead to death. Future studies may focus on improving the accuracy and reliability of these devices, as well as integrating them with IoT/IoMT platforms for real-time data analysis and the development of early-warning systems.

One of the major benefits of integrating wearable devices into clinical decision support systems is the ability to monitor patients at home without the need for intrusive medical devices and hospitalization; however, the main drawback is the amount of data generated by IoT/IoMT technologies. Researchers need to thoroughly explore the use of advanced data analytics techniques such as machine learning, deep learning, and artificial intelligence to analyze the large amounts of data collected from IoT/IoMT devices. In terms of data analytics, researchers can explore the development of predictive models that use historical patient data, genetic information, lifestyle factors, and IoT/IoMT data to assess an individual’s risk of developing CVD. These models could help to identify high-risk individuals for targeted interventions and preventive measures. As these techniques are expected to help in identifying patterns, correlations, and predictive models for the early detection and accurate classification of CVD, they also involve the collection and transmission of sensitive patient data. Therefore, future studies should also focus on developing robust security measures and privacy frameworks to protect data from unauthorized access and ensure compliance with privacy regulations.

Granular computing based on IoT/IoMT technologies has recently emerged, along with the advent of machine learning gaining significant attention in medical studies, with the potential to revolutionize various aspects of healthcare, including the diagnosis, treatment, and management of CVD. It allows data to be aggregated and grouped into granules that can be analyzed more efficiently and effectively. Granular computing can be viewed as a unified framework of theories and methods that utilize granularity in the problem-solving process. Granularity leads to information compression. Therefore, computing with granules instead of objects leads to faster computation times, making granular computing an important aspect of knowledge discovery and data mining [208].

Granular computing enables the representation of complex and heterogeneous CVD-related data collected from various IoT/IoMT devices, such as wearable sensors, remote monitoring systems, and health records. It also facilitates the fusion of multiple data sources in the IoT/IoMT ecosystem, such as physiological parameters, medical history, lifestyle factors, and environmental data. Granular computing can help to identify hidden patterns, correlations, and dependencies relevant to CVD through data clustering for medical data classification. It can provide great support in feature selection and extraction from the large amounts of data generated, improving the accuracy and efficiency of CVD classification and prediction models by reducing the dimensionality of the data and identifying the most informative features. These techniques support the development of algorithms that generate rules and decision models based on the extracted granularity, in order to provide insight into the relationships between CVD risk factors, symptoms, and outcomes. Granular computing enables continuous monitoring to assess the risk of developing CVD by analyzing granular data and identifying high-risk individuals. Continuous monitoring takes into account various factors such as age, gender, genetic markers, lifestyle habits, and physiological parameters. It provides personalized risk assessments that lead to personalized treatment and intervention strategies for individuals with CVD, taking into account individual patient characteristics. This helps to optimize treatment plans, recommend appropriate interventions, and support shared decision-making between patients and healthcare providers. It also allows for the analysis of large volumes of electronic health records and the classification of patients into different disease categories. CVD risk prediction enables targeted interventions and preventive measures to reduce the burden of CVD based on factors such as demographics, medical history, lifestyle data, genetic markers, and biomarkers.

In contrast, granular computing has been used in several machine learning approaches, such as disease diagnosis, risk prediction, treatment adaptation, prognosis detection, image analysis, decision support, precision medicine, biomarker discovery, and clinical trial design. Another approach is outlier detection, an important process for dealing with the outliers in a data set. More importantly, in disease prediction, the outliers are sometimes the most valuable data, as they indicate the values of those individuals with the disease.

With the continuous growth of technology, it is possible to classify, monitor, and predict chronic diseases, including CVD, in real-time and with high accuracy through the use of sensors and devices connected to the cloud. Recent proposals have focused on obtaining high accuracy with ensembles of algorithms or even new approaches to deal with the large amount of data generated; however, some of them have not placed an emphasis on privacy. They can classify and monitor vital signs, predict major risks, and send reminders or emergency alerts to healthcare providers, but they cannot handle the privacy of the sensitive and confidential data generated by IoT/IoMT devices in real-time. The data generated by IoT/IoMT devices contain details about an individual’s medical conditions, treatments, test results, and other personal identifiers. Privacy ensures that this information remains confidential and accessible only to authorized individuals. If the clinical decision support system can guarantee the privacy and confidentiality of the data generated, it will be a great tool for improving the remote monitoring of chronic diseases and helping to reduce the risk of death in even remote areas, where health care is expensive or not easily accessible, as will highly accurate devices or sensors for the tracking of vital signs.

Limitations of this study: First, we conducted a literature search in only four databases (PubMed, IEEE, Springer Link, and Science Direct). Second, we excluded studies that were not written in English, written before January 2016 and after May 2023, and articles other than JCR.

## 7. Conclusions

In this study, we examined the IoT/IoMT technologies and machine learning techniques used to detect, predict, or monitor CVD. We also identified several diseases that have been commonly detected or monitored. We classified 162 proposals obtained from the IEEE, PubMed, Science Direct, and Springer Link databases into the following categories: CVD detection using IoT/IoMT and CVD detection applying machine learning techniques. We found 78 articles in the former category and 84 in the latter. For these two categories, we extracted the following information: Study area, disease, data set, wearable device/smart device/medical device, machine learning approach, and results.

We noticed that the technologies and the machine learning approaches used in the proposals are continuously evolving, especially in terms of mobile systems, web applications, and frameworks. Some of the articles proposed a combination of techniques to achieve reliable performance in evaluation metrics (i.e., accuracy, precision, sensitivity, specificity, F1-score). Others used medical devices or commercial devices for the real-time monitoring of CVD. In these cases, we found that medical devices were a better recommendation for monitoring or detecting CVD due to their reliability.

Our review also revealed the type of diseases detected by wearable devices/smart devices/medical devices or machine learning approaches. The most-used wearable devices were medical devices, sensors, and microcontrollers. In some proposals, CVD or heart failure was detected as a common disease. In others, arrhythmia was the most commonly detected disease. The most-used machine learning or deep learning techniques were neural networks, including long short-term memory, convolutional neural networks, recurrent neural networks, artificial neural networks, bi-directional long short-term memory, and deep neural networks. The most used traditional classifiers included *k*NN, SVM, and random forest. However, after analyzing the results reported by the authors regarding the evaluation metrics, it is possible that some of the models were subject to over-fitting, due to the lack of data used for training and testing. We also identified the data sets most commonly used to train the models: UCI Cleveland, PhysioNet MIT-BIH, PhysioNet MIMIC-II, and Framingham. However, other proposals also used private data sets provided by hospitals.

The most important finding is the lack of public data sets focused on CVD. As more public CVD data sets become available, relevant machine learning and deep learning models could gain improved performance metrics, increasing the possibility of timely prediction of risk situations or even myocardial infarction. For example, after the COVID-19 pandemic, the demand for IoT/IoMT sensor devices in healthcare increased for the treatment and monitoring of critical patients, leading to better and more complete systems and frameworks for remotely monitoring chronic patients. Some commercial devices have recently begun to add features such as temperature sensing, measuring blood oxygen, and taking an ECG at any moment, allowing for caregivers to be alerted to any emergency detected. This leads to more smart options for the tracking of diseases.

From the results collected in this article, it seems clear that the detection and monitoring of CVD is possible. with the help of IoT/IoMT technology, it has recently become possible to monitor CVD in real-time and even alert caregivers in the case of an emergency. Thus, it is possible to triage the condition of the patient, recommend the nearest hospitals in the case of emergency, and send notifications to doctors and caregivers.

Finally, as the leading cause of death worldwide, it is of great importance to propose new machine learning approaches or better methods focused on CVD, in order to achieve good performance in terms of evaluation metrics and facilitate its real-time prediction.

## Figures and Tables

**Table 1:** Research questions related to the detection, prediction, or monitoring of CVD.

**RQ1**	What types of devices with IoT and IoMT technologies have been used to detect and predict cardiovascular disease using machine learning?
**RQ2**	What machine learning techniques have been used to detect and predict cardiovascular disease?
**RQ3**	What diseases were detected and predicted?

**Table 2:** PICOC template points.

**Population**	Formal publications on detection or prediction of cardiovascular disease
**Intervention**	Techniques, methods, or machine learning algorithms that are implemented through IoT or IoMT
**Comparison**	Comparison of technologies, methods, or algorithms implemented by IoT or IoMT
**Outcome**	Assessing the proposals analyzed for early detection of cardiovascular disease
**Context**	Technologies, techniques, and methods by means of computational mechanisms for the monitoring of people with cardiovascular diseases

**Table 3:** List of keywords used in the literature search.

**P**	Cardiovascular disease, heart disease, cardiovascular events, heart illness, heart condition
**I**	IoT, IoMT
**C**	Machine learning, deep learning, data mining
**O**	Early detection, detect, predict, monitor
**C**	Wearable devices, devices

**Table 4:** Top ten cited articles.

Year	Authors	Database	Name of the Journal	Number of Citations	Reference
2019	Mohan, S.; Thirumalai, G.; Srivastava, G.	IEEE	*IEEE Access*	888	[37]
2018	Yildirim, Ö. et al.	Science Direct	*Comput. Biol. Med.*	579	[38]
2020	Tuli et al.	Science Direct	*Future Gener. Comput. Syst.*	432	[39]
2018	Sannino, G.; De Pietro, G.	Science Direct	*Future Gener. Comput. Syst.*	351	[40]
2018	Kumar, P.M.; Gandhi, U.D.	Science Direct	*Comput. Electr. Eng.*	258	[41]
2019	Yildirim et al.	Science Direct	*Comput. Methods Programs Biomed.*	256	[42]
2020	Khan, M.A.	IEEE	*IEEE Access*	188	[43]
2019	Sellami, A.; Hwang, H.	Science Direct	*Expert Syst. Appl.*	158	[44]
2020	Khan, M.A.; Algarni, F.	IEEE	*IEEE Access*	153	[45]
2020	Lih et al.	Science Direct	*Artif. Intell. Med.*	146	[46]

**Table 5:** Type of disease in selected proposals using IoT or IoMT technologies.

A ${}^{1}$	B ${}^{2}$	C ${}^{3}$	D ${}^{4}$	E ${}^{5}$	F ${}^{6}$	Number of Proposals
[47,48,49,50,51,52,53,54,55,56,57,58,59,60,61,62,63,64,65,66,67,68,69,70,71]						25 (33.78%)
	[72,73,74]					3 (4.05%)
		[75,76,77]				3 (4.05%)
			[78,79,80,81,82,83,84,85]			8 (10.81%)
				[39,41,43,86,87,88,89,90,91,92,93,94,95,96,97,98,99,100,101,102,103,104,105,106,107,108,109,110]		29 (37.83%)
					[111,112,113,114,115,116,117,118,119,120,121]	10 (13.51%)

^1^ Abnormality detection/arrhythmia/atrial fibrillation. ^2^ Aortic stenosis/aortic valve. ^3^ Arterial stiffness/arterial. ^4^ Blood pressure/hypertension. ^5^ CVD/Heart disease/heart. ^6^ Others.

**Table 6:** IoT/IoMT-based abnormality and arrhythmia detection.

Wearable/Smart/Medical Device	Approach	Results
VA Processor/SoC, (Custom-made) [47]	Naive Bayes	Accuracy: 86%, Power consumption reduction: 62.2%
Electrodes (3 unipolar limb leads, 3 bipolar limb leads, 6 unipolar chest leads) [48]	Convolutional Neural Network	Accuracy: 98%, Sensitivity: 96%
Lenovo Smart ECG vest, (Lenovo Group Ltd., Beijing, China) [49]	Convolutional Neural Network	Accuracy: 86.3%
Arduino Uno, (Arduino, Scarmagno, Italy), Raspberry Pi 3B, (Raspberry Pi Foundation, Cambridge, UK) AD8232 ECG sensor (DFRobot, Shanghai, China), [50]	k-NN	Accuracy: 94.44%
Biomedical sensors, ARM processor, FPGA [51]	k-NN	Accuracy: 99%
Intelligent electrocardiograph device [52]	Neural network architecture based on deep learning	1st network Accuracy: 91%, 2nd network Accuracy: 100%, 3rd network Accuracy: 90%
AD8232 EKG sensor, (SparkFun Electronics, Niwot, CO, USA), Arduino board, (Arduino, Scarmagno, Italy), Jetson Nano microcomputer, (Nvidia Corporate, Santa Clara, CA, USA) [53]	Dynamic mode selected energy, adaptive window sizing, R location correction algorithm for detecting R-peaks with better efficiency	Accuracy: 99.94%, Sensitivity: 99.98%, Precision: 99.96% Specificity: 99.98% AUC: 99.89% Detection error rate: 0.06%
Raspberry Pi 3B (Raspberry Pi Foundation, Cambridge, UK) [54]	Fourier Transform, Convolutional Neural network (CNN)	Accuracy: 99.91% F1-Score: 95% Average inference time: 9 ms Maximun memory usage: 12 mb%
SensorTile (STEVAL-STLKT01V1), (STMicroelectronics, Grenoble, France), AD8232 (DFRobot, Shanghai, China), Raspberry Pi (Raspberry Pi Foundation, Cambridge, England, UK) [55]	Convolutional Neural network (CNN)	Accuracy: 97% Sensitivity: 96.92% Precision: 91.50% F1-Score: 94.89%
Raspberry Pi 4 (Raspberry Pi Foundation, Cambridge, UK) [56]	1D Convolutional Neural network (1D-CNN) GridSearch	Accuracy: 99.46%
Arduino Uno, (Arduino, Scarmagno, Italy), ATMEGA328P Microcontroller, (Microchip, AZ, USA) Raspberry Pi (Raspberry Pi Foundation, Cambridge, UK) [57]	Incremental Support vector Regression	Accuracy: 98.5% Sensitivity: 88% Precision: 90% Specificity: 99%
Sensor nodes [58]	Convolutional Neural network (CNN)	Accuracy: 95% Sensitivity: 94.63% Specificity: 94.63% ROC: 96.53%
Diagnosis and Tracking Shield, (Custom-made), ADS1298 (TX Instruments, Dallas, TX, USA), Raspberry Pi (Raspberry Pi Foundation, Cambridge, UK) [59]	Depth Convolutional Neural Network	Accuracy: 96.67% Sensitivity: 96.63% Specificity: 96.67%
ECG Machine [60]	Convolutional Neural network (CNN)	Accuracy: 99.12% Sensitivity: 100% Specificity: 99.12%
Smartphone device [61]	Convolutional Neural network (CNN)	Accuracy: 93%
MAC 5500 HD, (GE Healthcare, Chicago, IL, USA), MUSE v9, (GE Healthcare, Chicago, Illinois, USA) [62]	Convolutional Neural network (CNN)	Sensitivity: 88.50% Specificity: 88.54% Positive Predictive: 88.54% Negative Predictive: 88.54% F1-Score: 88.49%
Wearable sensors [63]	Convolutional Neural network (CNN), Artificial Bee Colony, Grey Wolf Optimizer	Accuracy: 94% Recall: 94.5% Precision: 96% Specificity: 95.4%
Noninvasive healthcare sensor, SkopEdge (Custom-made, India), Raspberry Pi, (Raspberry Pi Foundation, Cambridge, UK) [64]	Randon Forest	MIT-BIH Accuracy: 98.53% PTB Accuracy: 99% RF Accuracy: 98.68%
BH1790GLC (Rohm, Kyoto, Japan) [65]	Convolutional Neural network (CNN)	Sensitivity: 99.5% Specificity: 98.7% F1-Score: 99.1% Time: 19 s%
Sony Xperia Z-series, (Sony, Tokyo, Japan) [66]	Kernel SVM	Accuracy: 97.4%, Sensitivity: 93.8%, Specificity: 100%
AFE4403 (TX Instruments, Dallas, TX, USA) [67]	Linear Kernel SVM	TPR ${}^{1}$: 70.10%, TNR ${}^{2}$: 88.61%, Accuracy: 80.37%
Mason-Likar ECG 12-lead system (CardioCloud Medical Technology, Beijing, China) [68]	Deep Densely Connected Neural Network (DDNN)	Accuracy: 96.73%, Sensitivity: 96.67%, Specificity: 96.93%
KardiaMobile EKG Monitor (AliveCor Inc., CA, USA) [69]	Neural Network	AUC ${}^{3}$: 82.7%, Specificity: 74.9%
Sony Xperia Z1/Z5, (Sony, Tokyo, Japan), Philips IntelliBue MX40 (Philips, Amsterdam, Netherlands) [70]	Random Forest, XGBoost, Logistic Regression	AUC AFib ${}^{4}$: 98%, 98%, 96%, AUC ADHF${}^{5}$: 85%, 82%, 83%
ZYNQ Ultrascale ZCU106 FPGA, (Advanced Micro Devices, Inc., Santa Clara, CA, USA) [71]	1D Convolutional Neural network (1D-CNN)	Accuracy: 99.17%, Sensitivity: 97.03%, Specificity: 99.37%, Precision: 93.72%, F1-score: 97.90%

^1^ True positive rate. ^2^ True negative rate. ^3^ Data obtained from [124]. ^4^ Atrial fibrillation. ^5^ Acute decompensated heart failure.

**Table 7:** IoT/IoMT-based aortic stenosis detection.

Device	Machine Learning Approach	Results
3-axis MEMS accelerometer, Kionix KXRB5-2042, (Kionix, Inc., New York, USA), 3-axis MEMS gyroscope, Invensense MPU9150, (Invensense, Inc. San Jose, CA, USA) [72]	Decision Tree, Random Forest, Neural Network	Accuracy SCG ^1^: 94.79%, 95.94%, 93.54%, Accuracy GCG ^2^: 96.98%, 97.40%, 96.04%, Accuracy SCG + GCG ^3^: 96.98%, 98.96%, 97.08%
Non-implantable-mixed flow turbodynamic blood pump, Deltastream DP2 (Xenios AG, Helibronn, Germany) [73]	Cardiac output estimation pipeline utilizing a PIP sensor	Linear/quadratic discriminant analysis: Matthews correlation coefficient: 0.771 Sensititivity: 91.3% Specificity: 87.1%
GE Vingmed Ultrasound AS, (Norway Health Tech Horten, Norway) [74]	Two 3D Convolutional Neural Nerwork	LV function detection Accuracy: 86% AV regurgitation detection Accuracy: 83%

^1^ Seismo-cardiography: measurement of the linear acceleration components of the chest wall induced by the heartbeat. ^2^ Gyro-cardiography: recording of heart-induced rotational vibrations of the chest wall in the form of angular speed. ^3^ SCG and GCG signals can be acquired by placing a microelectromechanical system (MEMS) inertial measurement unit (IMU) on the chest wall.

**Table 8:** IoT/IoMT-based arterial stiffness detection.

Wearable/Smart/Medical Device	Machine Learning Approach	Results
OMRON BP-203RPE III, (OMRON Industrial Automation, Kyoto, Japan) [75]	Multiple Linear Regression, Back Propagation Neural Network	Accuracy: 89%, 94%
Heart rate monitoring, respiratory sensor, optical sensor [76]	PCA, Deep Belief Networks Long Short-Term Memory	Accuracy: 88.42% Sensitivity: 85.13% Specificity: 85.54%
Bio-sensors [77]	Diagnostic system based on implanted devices and nano-nodes circulating in the cardiovascular system	Diagnosis of blocked artery in 3 h, and medication released by another 3 h

**Table 9:** IoT/IoMT-based blood pressure and hypertension detection.

Wearable/Smart/Medical Device	Machine Learning Approach	Results
Accelerometer, GPS, ECG, Blood Pressure Monitor [78]	Multilayer Perceptron, Decision Tree J48, Decision Table, Radial Basis Function, Bayes Network	Accuracy for three different types of patient Accuracy MLP: 91.46%, 95.54%, 90.75%, Accuracy J48: 99.14%, 99.78%, 99.1%, Accuracy DTable: 95.91%, 97.08%, 96.33%, Accuracy RBF: 81.52%, 83.95%, 84.78%, Accuracy BN: 86.58%, 95.11%, 88.55%
Impedance cardiography sensor, (Custom-made, India) [79]	Auto-adaptive algorithm based on Impedance Cardiography signals for non-invasive, cuffless, continous monitoring of blood pressure and heart rate	Systolic BP: ±2.33 mmHg Diastolic BP: ±3.60 mmHg Heart rate: ±2.88 beats
ADS1299EEG-FE, (TX Instruments, Dallas, TX, USA), AFE4490SPO2, (TX Instruments, Dallas, TX, USA), MSP430F55291PN, (TX Instruments, Dallas, TX, USA) [80]	SVM, Dynamic Time Warping (DTW), K-medoids clustering	ME ${}^{1}\pm$ STD ${}^{2}$: 0.8±2.7 BPM ${}^{3}$, MAE ${}^{4}$: 1.8 BPM, RMSE ${}^{5}$: 2.8 BPM For HR estimation
Ring PPG, Accelerometer, ZigBee, (Custom-made device, Taiwan) [81]	MIL (Multiplate instance learning algorithm)	Accuracy Standard Deviation of all RR (NN) intervals: 85.74%, Specificity: 83.33%, Precision: 92.11%, Sensitivity: 86.42%
Raspberry Pi 2, (Raspberry Pi Foundation, Cambridge, UK) [82]	Random Forest, Decision Tree, SVM, AdaBoost	SBP ${}^{6}$ RMSE ${}^{5}$: 3.2±0.7 mmHg, DBP ${}^{7}$ RMSE ${}^{5}$: 2.2±0.7 mmHg, SBP ${}^{6}$ MAE ${}^{4}$: 4.4±1.0 mmHg, DBP ${}^{7}$ MAE ${}^{4}$: 2.9±1.2 mmHg
Pulse oximeter, (Arduino, Scarmagno, Italy) [83]	k-NN, SVM, Decision Tree, Neural Network	10 fold cross k-NN Precision: 91%, SVM Precision: 96%, DT Precision: 95%, NN Precision: 96%, LOOCV ${}^{8}$ k-NN Precision: 90%, SVM Precision: 93%, DT Precision: 94%, NN Precision: 95%
CMS50FW Pulse Oximeter, (Contec Inc., Qinhuangdao, China) Finometer MIDI Model II, (Finapres Medical Systems B.V., Amsterdam, The Netherlands) [84]	SVM	MAE ${}^{4}$: systolic 0.043 mmHg, diastolic 0.011 mmHg, mean blood pressure 0.008 mmHg
Mindray N12, (Mindray, Shenzhen, China) [85]	Residual Network Long Short-Term Memory Network (Res-LSTM)	SBP${}^{6}$ Mean difference ± Standard deviation accuracy: −0.2 ± 5.82 mmHg, Mean Arterial Pressure Mean difference ± Standard deviation accuracy: −0.57 ± 4.39 mmHg DBP ${}^{7}$, Mean difference ± Standard deviation accuracy: −0.75 ± 5.62 mmHg

^1^ Mean error. ^2^ Standard deviation. ^3^ Beats per minute. ^4^ Mean absolute error. ^5^ Root mean square error. ^6^ Systolic blood pressure. ^7^ Diastolic blood pressure. ^8^ Leave-one-out cross-validation.

**Table 10:** IoT/IoMT-based cardiovascular and heart disease detection.

Wearable/Smart/Medical Device	Approach	Results
Zephyr HxM heart rate/BioHarness (Zephyr Technology Corporation Annapolis, Maryland, USA) [86]	Pan Tompkins algorithm	10 times faster and consumed 90% less energy
Holter [87]	Decision Tree, SVM	ECG accuracy: 90.77%, AP clustering accuracy: 87.40%, Action recognition accuracy: 93.36%, AP clustering accuracy: 94.49%
Raspberry Pi 2, (Raspberry Pi Foundation, Cambridge, England, UK), 2014 Motorola Moto G, (Motorola Mobility LLC Chicago, IL, USA) [88]	SVM, Multilayer Perceptron (MLP)	Accuracy: 71.30%, 77.50%, Sensitivity: 45.5%, 60.37%, Specificity: 76.21%, 61.03%
Raspberry Pi 3B, (Raspberry Pi Foundation, Cambridge, UK) [39]	Deep Neural Network, Bagging Classifier	Accuracy 1 edge node: 78%, 2 edge node: 78%, 3 edge node: 72%, 4 edge node: 74%, 5 edge node: 74%
Wearable watch [89]	Boltzmann Deep Belief Neural Network (HOBDBNN), Genetic Algorithm-Based Trained Recurrent Fuzzy Neural Networks (GA-TRFNN), Swarm Optimized Convolutional Neural Network-Support Vector (SCNN-SVM), Particle Optimized Feed Forward Back Propagated Neural Network (PFFBPNN), Particle Swarm Optimized Radial Basis Function Network (PSRBFN)	Accuracy: 99.03%
Distance 2Go radar, INFINEON entry-level kit (Infineon Technologies AG, Munich, Germany) [90]	Long Short-Term Memory (LSTM)	Train loss: 00086, Valid loss: 0.0054
SensEcho, (Beijing SensEcho Science & Technology Co, Ltd., Beijing, China) [91]	Bidirectional Long Short- Term Memory (BI-LSTM)	Bradycardia sensitivity: 92.86%, Bradycardia specificity: 99.92%, Bradycardia precision: 85.53%, Tachycardia sensitivity: 81.44%, Tachycardia specificity: 99.80%, Tachycardia precision: 84.24%
Wearable sensor for Smart Healthcare Monitoring System (SHMS) [98]	SVM, k-NN, Naive Bayes, Decision Tree	Accuracy: 92%, 72%, 83%, 75%, F1-score: 85%, 72%, 84%, 76%
Smartwatch, OMRON HeartGuid bp8000m, (OMRON Industrial Automation, Kyoto, Japan), AD8232 SparkFun Single Lead Heart Rate Monitor, (SparkFun Electronics, Niwot, CO, USA) Raspberry Pi, (Raspberry Pi Foundation, Cambridge, UK) [43]	Modified Deep Convolutional Neural Network (MDCNN)	Accuracy: 98.2%
Arduino Uno, (Arduino, Scarmagno, Italy), Finger tip heart rate sensor	Linear Regression	————
Wearable IoT device [41]	Logistic Regression	Sensitivity Respiratory Rate: 92.06%, Heart rate: 72.38%, Blood pressure SR ^1^: 85.71%, Blood pressure DR ^2^: 48.25%, Body temperature: 82.54%, Blood sugar fasting: 60.63%, Blood sugar post-meal: 25.8%
IR Plethysmograph Velcro Strap, MLT 1020 PPG, (AD Instruments, Sydney, Australia), Bio-amplifier, Dual Bio-AMP-FE 232, (AD Instruments, Sydney, Australia), DAQ, Power Lab 8/35, ML135, (AD Instruments, Sydney, Australia) [92]	Deep Neural Network	Accuracy: 80%, Recall: 75%, Precision: 73%, F1-Score: 78%,
Raspberry Pi, (Raspberry Pi Foundation, Cambridge, UK) [93]	Deep Neural Network (DNN), Logistic Regression, Random Forest	LR F1-Score: 84% LR Precision: 87% LR Accuracy: 83% LR Recall: 82% LR Specificity: 84% RF F1-Score: 85% RF Precision: 86% RF Accuracy: 82% LR Recall: 83% RF Specificity: 83%
Sense O’Clock smartwatch, (Custom-made device, Australia) [94]	SVM, k-NN, XGBoost, Support Vector Regression	RF Accuracy: 99% k-NN Accuracy: 99.3% XGBoost Accuracy: 98.56%
IoT-enabled WPM devices [95]	Decision Tree, One-dimensional convolutional neural network-long short-term memory(1D CNN-LSTM)	PPG-BP dataset DT Accuracy: 99.5% PPG-DaLiA CNN Accuracy: 97.56%
Polar H7 heart rate monitor, (Polar Electro Inc., Bethpage, NY, USA), Actigraph data, ACTi Graph wGT3X-BT, (ACTi Graph LLC, Pensacola, FL, USA) [96]	Linear Regression, SVM, k-NN, LSTM, Decision Tree, Random Forest	k-NN regressor and LSTM performed the worst, with SI scores of 41.36% and 34.15%, respectively
MOYO mobile platform, (Custom-made platform), Omron M7 (OMRON Industrial Automation, Kyoto, Japan), Jawbone UP3, (Jawbone Health, San Francisco, CA, USA) [97]	Electronic cohort study, HealthTech Events	The research team collected 13 prototypes, consisting of 297 screens
ECG sensor, temperature sensor Electroencephalagram sensor, electromyography sensor, oxygen level sensor, respiration rate sensor, glucose level [99]	Deep Learning	Accuracy: 89.98% Precision: 88.8% Specificity: 89.72% Recall: 89.72% F1-Score: 89.96%
AD8232 SparkFun Single Lead Heart Rate Monitor, (SparkFun Electronics, Niwot, CO, USA), Arduino Uno, (Arduino, Scarmagno, Italy) [100]	Random Forest	Accuracy: 88.10% Precision: 93.75% Recall: 78.95% F1-Score: 85.71%
On-sensor, (Custom-made, Buffalo, NY, USA) [101]	Mixed-signal neural network and reservoir-computation (RC-NN)	Heart Disease Accuracy: 86.8% Sensitivity: 83% Specificity: 89%
ESP8266 NodeMCU Wi-Fi Devkit, (Arduino, Scarmagno, Italy), MAX30102 board, (DFRobot, Shanghai, China), DS18b20 sensor, (DFRobot, Shanghai, China), DHT22 sensor, (DFRobot, Shanghai, China), AD8232 ECG sensor (DFRobot, Shanghai, China) [102]	Portable IoT-based health monitoring system	Error percentage Body temperature: 2.67% Heart rate: 2.04% SpO${}_{2}$: 1.58%
Server nodes, smartphone nodes [103]	Adaptive multiple dictionary learning-based joint compressive sensing for MECG compression	Percent root mean-squared difference: 3.942
Medtonic sensor, (Medtronic, Minneapolis, USA), Heartbeat sensor, (Sunrom Electronics, Ahmedabad, Gujarat) [104]	Healthcare monitoring system based on IoMT and cloud-fog environment	Accuracy: 97.32% Recall: 97.58% Precision: 97.16% F1-Score: 97.37% Specificity: 96.87%
ECG electrodes, Microcontroller PIC24FJ64GB002 (Microchip Technology Inc., Chandler, AZ, USA) [105]	Probabilistic Neural Network	Heart disease Accuracy: 60.98% Precision: 58.32% Sensitivity: 68% Specificity: 55.1%
Holter monitor [106]	Bi-directional Short Term Memory Network	Accuracy: 98.7% Precision: 99.1% Recall: 99.9%
Ballistocardiogram sensor, (Custom-made, Wuhan, China), Polysomnography equipment (SOMNO medics GmbH, Randersacker, Germany) [107]	Ballistocardiogram (BCG)-based system	Median error of 4.4 ms
Pulse Express Pulse-Ox, & Heart Rate Sensor (ProtoCentral Electronics Pvt Ltd., Bengaluru, Karnataka, India), Arduino 1010 WIFI MKR, (Arduino, Scarmagno, Italy) [108]	Automatic multiscale-based peak algorithm	Accuracy: 98.7%
ECG sensor, (Custom-made) [109]	Residual Convolutional Neural Network	Accuracy: 99.58% Precision: 98.5% Recall: 99% AUC: 99.8%
SmartCardia INYU, (SmartCardia Inc., Lausanne, Switzerland) [110]	Convolutional Neural Network (CNN)	Accuracy: 99.2% Precision: 99.5% Specificity: 99.4% Sensitivity: 99.2%

^1^ Systolic rate. ^2^ Diastolic rate.

**Table 11:** IoT/IoMT-based detection of different diseases.

Wearable/Smart/Medical Device	Machine Learning Approach	Results
Smartphone Samsung Galaxy Young (Samsung Electronics Co., Yeongtong-gu, Suwon-si, South Korea.) [111]	SVM	Accuracy: 97.74%, Precision: 92.21%
EEG sensors, ECG sensors, accelerometer, gateway module [112]	Random Forest	Accuracy: 83.35%, Precision: 91.32% Recall: 91.32%, F1-Score: 65%
SmartCardia INYU, (SmartCardia Inc., Lausanne, Switzerland) [113]	Random Forest	Sensitivity: 87.95%, Specificity: 78.82%
Electronic nose: 1 humidity sensor 18 electrochemical gas sensors [114]	SVM	Accuracy: 97.19%, Sensitivity: 93.37% Specificity: 99.07%
Network on body-area sensor (BAS) Raspberry Pi 3B+, (Raspberry Pi Foundation, Cambridge, UK) [115]	Deep Neural Network (DNN)	Accuracy: 90%
Smart device sensors [116]	ResNet-9, federated semi-supervised learning (FSSL)	Accuracy: 95.9%
Photoplethysmography sensor, temperature sensor, accelerometer 12C slave sensor, microcontroller 12C master, (Custom-made, Miami, USA) [117]	Long Short-Term Memory (LSTM)	Root mean square: 0.07% Accuracy: 99.5%
Motion sensor, ECG sensor EMG sensor, Foot sensor [118]	Long Short-Term Memory (LSTM)	Accuracy: 98.99%
Raspberry Pi 3B, (Raspberry Pi Foundation, Cambridge, UK) NVidia Jetson Nano, (NVidia, Santa Clara, CA, USA) [119]	Deep Neural Network (DNN)	Accuracy: 99.8%
ECG sensors [120]	R-peak detection algorithm	Reduction of the data dropout rate, by average of 21.09% Number of R-peak detections increased by 15.33% compared to the existing classification system
NRF52 cortex ARM M4F microcontroller (NRF52DK), (Nordic semiconductor, Trondheim, Norway) [121]	Artificial Neural Network (ANN)	INCART Accuracy: 93% INCART Sensitivity: 88% INCART Specificity: 94% INCART Precision: 67%

**Table 12:** Types of disease detected in selected proposals by applying machine learning techniques.

A ${}^{1}$	B ${}^{2}$	C ${}^{3}$	D ${}^{4}$	E ${}^{5}$	F ${}^{6}$	Number of Proposals
[38,40,42,44,129,130,131,132,133,134,135,136,137,138,139,140,141,142,143,144,145,146,147,148,149,150,151,152,153,154,155,156,157]						33 (39.28%)
	[158,159,160,161,162,163,164,165,166,167]					10 (11.90%)
		[37,45,46,168,169,170,171,172,173,174,175,176,177,178,179,180,181,182,183,184,185,186,187,188]				25 (28.57%)
			[189,190,191,192,193,194]			6 (7.14%)
				[195,196,197,198]		4 (4.76%)
					[199,200,201,202,203,204,205]	7 (8.33%)

^1^ Abnormality detection/arrhythmia/atrial fibrillation. ^2^ Blood pressure/hypertension. ^3^ CVD/Heart disease. ^4^ Myocardial infarction. ^5^ Coronary heart/Coronary artery disease. ^6^ Others.

**Table 13:** Abnormality and arrhythmia detection using machine learning techniques.

Data Set	Approach	Results
MIT-BIH (MLII) [38]	Convolutional Neural Network (CNN)	Accuracy: 91.33%
MIT-BIH Arrhythmia [40]	Deep Neural Network	Accuracy: 99.68%, Sensitivity: 99.48%, Specificity: 99.83%
MIT-BIH [42]	Long Short-Term Memory (LSTM)	Accuracy: 99%
MIT-BIH Arrhythmia [44]	Convolutional Neural Network (CNN)	Intra-patient Accuracy: 99.79%, Positive productivity: 97.71%, Sensitivity: 94.65%, Specificity: 99.36% Inter-patient Accuracy: 88.34%%, Positive productivity: 48.25%, Sensitivity: 90.90%, Specificity: 88.51%
MIT-BIH Arrhythmia [129]	SVM	Accuracy (Gaussian linear, Polynomial kernel): 91.69%, 88.14%, 88.74%
MIT-BIH Arrhythmia, Robust Detection of Heart Beats in Multimodal Data (RDHBMD) [130]	Linear-Kernel SVM	Supraventricular ectopic beat (SVEB) F1-score: 83%, Sensitivity: 79.3%, Specificity: 99.6%, Positive predictive value: 88.3% Ventricular ectopic beat (VEB) F1-score: 92%, Sensitivity: 92.8%, Specificity: 99.4%
MIT-BIH MITDBA, MIT-BIH FVADB, Ventricular Tachyarrhythmia from Creighton University (VTADB) [131]	SVM	Accuracy: 98.9%, Sensitivity: 99.08%, Specificity: 97.11%
MIT-BIH Atrial Fibrillation, MIT-BIH Arrhythmia [132]	Wavelet Transform, Bacterial-Foraging Optimization (BFO), Particle Swarm Optimization (PSO)	AFib ^1^-WT ^2^ accuracy: 99.1%, MI ^3^-SVM accuracy: 98.9%, BBB ^4^-SVM accuracy: 99.3%
MIT-BIH Arrhythmia [133]	Multi-scale Fusion-Convolutional Neural Network	Accuracy; 98%, Sensitivity: 96.17%, Specificity: 96.38%
CPSC_2018, PhysioNet/CinC_2017 [134]	End-to-End Deep Multi-Scale Fusion Convolutional Neural Network (DMSFNet)	F1-score CPSC: 82.8%, F1-score CinC: 84.01%, Accuracy CPSC: 83%, Accuracy CinC: 85%
MIT-BIH Atrial Fibrillation Database (AFDB) [135]	Lead Convolutional Neural Network (LCNN)	AUC: 93.17%, Sensitivity: 98.51%, Specificity: 98.26%
PhysioNet Atrial Fibrillation [136]	Long Short-Term Memory (LSMT)	Accuracy: 98.15%
MIT-BIH [137]	Long Short-Term Memory (LSMT)	Accuracy: 94%, AUC: 96.58%, Precision: 95%, Sensitivity: 95%
MIT-BIH Atrial Fibrillation [138]	Linear regression, k-NN, CART, SVM, Random Forest, XGBoost	Random Forest Accuracy: 95.47%, Sensitivity: 94.54%, Specificity: 96.40%, Precision: 96.55%, F1-score: 95.56%
Long-Term Atrial Fibrillation Database (LTAFDB) [139]	Random Forest	AUC/AP (Average Precision): 50% compression: 91%, 90%, 75% compression: 92%, 91%, 95% compression: 82%, 91%
MIT-BIH Arrhythmia [140]	Genetic Algorithm, Deep Neural Network, k-NN	Accuracy: 98%
MIT-BIH Arrhythmia [141]	Linear Discriminant Analysis	Normal Sensitivity: 93.7% Precision: 99.2% Supraventricular ectopic beat Sensitivity: 89.7% Precision: 36.8% Entricular ectopic beat Sensitivity: 87.9% Precision: 93.9%
MIT-BIH Arrhythmia, The European Society of Cardiology ST-T, Boston’s Beth Israel Deaconess Medical Center [142]	Convolutional Bidirectional Long Short-Term Memory Neural Networks Time adaptive Convolutional Neural Networks	Congestive heart failure events Accuracy: 100% Arrhtythmia events Accuracy: 97.9% Sudden cardiac deaths Accuracy: 100%
MIT-BIH Arrhythmia, Creighton University Ventricular Tachyarrhythmia, MIT-BIH Atrial Fibrillation, MIT-BIH Malignant Ventricular Ectopy [143]	Convolutional Neural Network (CNN) (AlexNet, VGG16, VGG19)	First stage Accuracy: 98.41% Second stage Accuracy: 95.3%
PhysioNet 2017 [144]	Convolutional Neural Network (CNN), SVM	F1-Score: 84.19% Precision: 81.65% Recall: 75.88%
ECG Rhythm [145]	Deep Neural Network, k-NN, SVM, Random Forest, Naive Bayes, GBoost, AdaBoost, Decision Tree, Multilayer Perceptron	RF Accuracy: 98% RF Sensitivity: 97.69% RF Specificity: 99.34% RF Precision: 97.77% RF F1-Score: 97.72%
MIT-BIH, MIT-BIH NSR, BIDMC [146]	Hybrid Deep CNN	Accuracy: 98.75% Specificity: 99% Sensitivity: 98.18% Time: 0.15 seg
MIT-BIH Arrhythmia [147]	One-Dimensional Neural Network (1D-CNN)	Accuracy: 98.35% Precision: 99.36% Sensitivity: 98.18%
MIT-BIH Arrhythmia [148]	Convolutional Neural Network (CNN)	Accuracy: 98.82% Sensitivity: 93.14% Specificity: 94.73% F1-Score: 93.52%
MIT-BIH Arrhythmia [149]	Convolutional Neural Network (CNN)	Accuracy: 99.4% Precision: 97.6% Specificity: 99.7% Sensitivity: 97.1%
MIT-BIH Arrhythmia PTB Diagnostic ECG [150]	Deep Learning and fuzzy clustering (Fuzz-ClustNet)	Accuracy: 98.66% Precision: 98.92% Recall: 93.88% F1-Score: 96.34%
MIT-BIH Arrhythmia [151]	Deep Residual Convolutional Neural Network	Normal segments Sensitivity: 94.54% Precision: 93.33% Specificity: 80.80% Supraventricular segment Sensitivity: 35.22% Precision: 65.88% Specificity: 98.83%
MIT-BIH Arrhythmia [152]	Feedforward and recurrent deep neural networks	Accuracy: 99.46% Specificity: 99.57% Sensitivity: 99.46% Precision: 98.26% F1-Score: 97.63%
MIT-BIH Arrhythmia [153]	Sequential Artificial Features	F1-Score: 98.96% Precision: 98.93% Sensitivity: 99%
CinC2017, ICBEB2018 [154]	Densely Connected Convolutional Network	CinC2017 F1-Score: 83.10% ICBEB2018: 82.60%
PLA General Hospital, CPSC 2018, MIT-BIH Arrhythmia [155]	Convolutional Neural Network (CNN)	F1-Score: 99.57% Accuracy: 99.89% Precision: 99.28% Specificity: 99.93% Sensitivity: 99.86%
MIT-BIH Arrhythmia [156]	Convolutional Neural Network (CNN)	Accuracy: 99.01% Sensitivity: 99.11% Precision: 99.02%
Atrial Fibrillation Prediction PyshioNet [157]	SVM, k-NN	Sensitivity: 98.8% Specificity: 96.7% Accuracy: 97.7%

^1^ Atrial fibrillation. ^2^ Wavelet transform. ^3^ Myocardial infarction. ^4^ Bundle branch block.

**Table 14:** Detection of blood pressure and high blood pressure using machine learning techniques.

Data Set	Approach	Results
PhysioNet [158]	ANOVA, Chi-squared, Decision Tree, Random Undersampling Boosting (RUSBOOST)	Accuracy: 97.08%, Precision: 81.25%, F1-score: 86.67%
MIMIC-II Database [159]	Nonlinear Auto-regressive Model with Exogenous Input (NARX), ANN Perceptron	Mean Absolute Error (MAE) 10 beat ECG: 3.91±4.90, PPG: 2.59±3.21, ECG+PPG: 3.05±3.81
MIMIC-II Database [160]	Recurrent Neural Network (RNN), Long Short-Term Memory (LSTM), Bidirectional Long Short-Term Memory (BI-LSTM), Grated Recurrent Units (GRU)	Averages using 1, 2, and 3 hidden layers (RMSE) RNN: 3.025, 3.065, 3.055 LSTM: 3.106, 3.074, 3.06 BI-LSTM: 3.101, 3.069, 2.840 GRU: 3.077, 3.084, 3.061
MIMIC-II Database [161]	Convolutional Neural Network (CNN)	Large db MAE Systolic BP: 3.70 Diastolic BP: 2.81 Small db MAE Systolic BP: 1.37 Diastolic BP: 0.93
UCI Machine Learning Repository [162]	Multi-scale fusion neural networks and multi-task learning	Mean and std Systolic BP: 0.97±8.87 Diastolic BP: 0.55±4.23
MIMIC-II Database [163]	Deep Learning (BiLSTM-At)	Classification: 92.19% Normal Subjects Accuracy Systolic BP: 2.815 mmHG and 1.876 mmHG AF Subjects Accuracy Systolic BP: 3.024 mmHG and 1.334 mmHG CA Subjects Accuracy Systolic BP: 4.444 mmHG and 2.549 mmHG
MIMIC-II Database [164]	Deep Learning	Diastolic BP Root mean square error: 1.17 Mean absolute error: 1.04 Systolic BP Root mean square error: 1.06 Mean absolute error: 1.02
MIMIC-II Database [165]	Recurrent Neural Network with bidirectional connections	Systolic BP 7-feature set 2.9±3.94 Diastolic BP 1.31±1.76
MIMIC-III Database [166]	BiConvLSTM	MAE: 2.29 Mean: 0.075
MIMIC-II Database [167]	Deep Learning	Systolic BP Accuracy: 91.44% Sensitivity: 70.17% Specificity: 94.20% F1-Score:70.07% Diastolic BP Accuracy: 94.66% Sensitivity: 83.10% Specificity: 94.88% F1-Score: 84.67%

**Table 15:** Detecting cardiovascular and heart disease using machine learning techniques.

Data set	Approach	Results
Cleveland, Hungarian, Long-beach VA, Switzerland (UCI) [168]	SVM, Naive Bayes, Random Forest, Multilayer Perceptron	Accuracy: 98%
Cleveland (UCI) [37]	Hybrid Random Forest with a Linear Model	Accuracy: 95.87%
Hungarian HD (UCI) [169]	Deep Learning Modified Neural Network (DLMNN)	Security: 95.87%
Statlog Cleveland (UCI) [170]	Density-Based Spatial Clustering of Application with Noise (DBSCAN)/SMOTE-ENN/XGBoost	STATLOG dataset Accuracy: 95.90 ± 5.55, Precision: 97.14 ± 5.71, Sensitivity: 94.67 ± 11.08, Cleveland dataset Accuracy: 98.40 ± 3.21, Precision: 98.57 ± 4.29, Sensitivity: 98.33 ± 5.00
Cleveland (UCI) [45]	Modified Salp-Swarm Optimization-Adaptative Neuro-Fuzzy Inference System (MSSO-ANFIS)	Accuracy: 99.45%, Precision: 96.54%
PTB Diagnostic ECG, Fantasia Database, St. Petersburg Institute of Cardiological Technics 12-lead Arrhythmia Database, PTB Diagnostic ECG Database, BIDMC Congestive Heart Failure Database [46]	Convolutional Neural Network (CNN), Long Short-Term Memory (LSTM)	Accuracy: 98.51%, Specificity: 97.89%, Sensitivity: 99.30%, Positive predictive value: 97.33%
PhysioNet [171]	Particle Swarm Optimization (PSO), Twin Support Vector Machine (TSVM)	Accuracy: 96.68%
Framingham and Hungarian Kaggle, Health Dataset USA Health site [172]	Beetle Swarm Optimization-Adaptive Neuro-fuzzy inference system (BSO-ANFIS)	BSO-ANFIS of heart disease classification Accuracy: 99.1%, Precision: 99.37%, Specificity: 99.4%, Sensitivity: 99.21% BSO-ANFIS of multi-disease identification Accuracy: 96.08%, Precision: 98.63%
MIT-BIH Arrhythmia, BIDMC Congestive Heart Failure, MIT-BIH Normal Sinus Rhythm	Deep Neural Network	Accuracy: 99%
Cleveland, South Africa, Z-Alizadeh Sani, Framingham, Statlog [173]	Deep Belief Network	Cleveland Accuracy: 89.2% South Africa Accuracy: 89.5% Z-Alizadeh Sani Accuracy: 89.7% Framingham Accuracy: 90.2% Statlog cardiac disease Accuracy: 91.2%
Computing in Cardiology Challenge [174]	Neural Network	Accuracy: 97%
UCI Machine Learning Repository [175]	Artificial Neural Network (ANN)	Accuracy: 95.78% Precision: 95.2% Recall: 95.2% Equal rate of error: 4.32%
Dalian Medical University and Northeastern University [176]	Three Decision Tree-based multilabel learning methods	F1-score: 86.73% AUC: 90.80% Accuracy: 92.72%
Statlog, Cleveland, Hungary [177]	Deep Bidirectional Long Short-Term Memory with Elliptic Curve Cryptography dependent Diffi-Huffman algorithm	Accuracy: 97.53% Sensitivity: 97.93% Specificity: 97.52% F1-Score: 7.65%
Cleveland [178]	Big data processing Apache Spark Apache Kafka	Accuracy: 92.05% Sensitivity: 88.10% Specificity: 95.65%
UCI public repository [179]	Sine Cosine Weighted k-NN	Accuracy: 92.13% Precision: 88.21% Recall: 93.27% F1-Score: 90.60% RMSE: 0.1115
MAHNOB-HCI, MMSE-HR, UBFC-Rppg, VIPL-HR [180]	Convolutional Neural Network (CNN)	VIPL-HR Accuracy: 90% MAE: 5.23 RMSE: 7.21
Kaggle repository [181]	Recursive feature elimination based Gradient Boosting (RFE-GB)	Accuracy: 88.8% Precision: 88.8% Recall: 85% F1-score: 83% AUC: 84% MSE: 0.20 RMSE: 0.44
Cleveland [182]	Multi-layer Perceptron for Enhanced Brownian Motion based on Dragonfly Algorithm	Accuracy: 97.47% Sensitivity: 98.92% F1-score: 96.45% Specificity: 96.47% Kappa: 96.75%
Chapman University and Shaoxing People’s Hospital, China Physiological Signal Challenge [183]	Deep Learning System	F1-score: 97.18% Precision: 97.36% Recall: 97.03% Accuracy: 98.73%
UCI Heart Disease [184]	Stacking Classifiers Model	Accuracy: 91.8% Precision: 92.6% Sensitivity: 92.6% Specificity: 91%
Breast Cancer, Heart Disease, Pima of UCI repository [185]	Multi-layer Perceptron	Heart Disease prediction with MLP-AOA-AE Accuracy: 83.90% Precision: 84.69% F1-score: 83.84% Recall: 83.90%
Pulsewatch, UMMC Simband Stanford University’s PPG, MIMIC-III [186]	Ensemble based feature selection	MIMIC-III Accuracy: 99% Sensitivity: 83% Specificity: 98% UMMC Accuracy: 94% Sensitivity: 95% Specificity: 91%
CapnoBase, MIMIC-II [187]	Growing Multilayer Network	CapnoBase Sensitivity: 98.49% Precision: 98.60% F1-score: 98.55% MIMIC-II Sensitivity: 96.01% Precision: 98.35% F1-score: 97.17%
Mindray, MIMIC [188]	Knowledge Distillation Strategies	Estimation error Systolic BP: 0.02±5.93 mmHG Diastolic BP: 0.01±3.87 mmHG

**Table 16:** Detecting myocardial infarction using machine learning techniques.

Data Set	Machine Learning Approach	Results
Physikalisch-Technische Bundesanstalt (PTB) diagnostic ECG, AF Classification [PhysioNet] [189]	Convolutional Neural Network–Long Short-term memory (CNN-LSTM)	Sensitivity: 92.4%, Specificity: 97.7%, PPV ^1^: 97.2%, F1-score: 94.6%
Physikalisch-Technische Bundesanstalt [190]	Convolutional Neural Network	Accuracy: 98.13%, Sensitivity: 98.19%, Specificity: 98.09%
MIT-BIH, Electrocardiogram Vigilance with Electronic data Warehouse (ECG-ViEW II) [191]	Convolutional Neural Network (CNN), Recurrent Neural Network, XGBoost	Accuracy: 89.8%, 84.6%, 97.5%, Sensitivity: 93.2%, 78%, 93.5%, Specificity: 88.1%, 87.8%, 99.4%, F1-score: 89%, 82.8%, 97.1%, AUROC: 90.7%, 82.9%, 96.5%
Medical records from the hospital information system [192]	Random Forest	AUC: 85%, Accuracy: 82%
MIT PhysioNet PTB diagnostic ECG [193]	k-NN	Accuracy: 99.96% Sensitivity: 99.96% Specificity: 99.95%
Physikalisch-Technische Bundesanstalt diagnostic ECG [194]	Long Short-Term Memory (LSTM)	Accuracy: 89.56% Recall: 91.88% Specificity: 80.81%

^1^ Positive predictive value.

**Table 17:** Detecting coronary artery disease and coronary heart disease using machine learning techniques.

Data Set/Study	Machine Learning Approach	Results
——— [195]	Logistic regression, Elastic Net, SVM, Random Forest, XGBoost	AUC CAD ^1^ classification LR: 0.79 ± 0.03, EN: 0.90 ± 0.03, SVM: 0.82 ± 0.03, RF: 0.83 ± 0.03, XGBoost: 0.85 ± 0.03 AUC LVEDP ^2^ classification LR: 0.77 ± 0.05, EN: 0.89 ± 0.03, SVM: 0.76 ± 0.04, RF: 0.73 ± 0.05, XGBoost: 0.81 ± 0.04, Sensitivity CAD EN: 80%, Specificity CAD EN: 80%, Sensitivity LVEDP EN: 91%, Specificity LVEDP EN: 81%
Framingham Heart Study [196]	Multilayer Perceptron	Accuracy: 96.50%, Sensitivity: 91.90%, Specificity: 98.28%
Cardiovascular Disease Dataset [197]	k-NN, Bagging, Binary Logistic Classification, Naive Bayes, Boosting	Bagged Decision Accuracy: 73.9% Gradient Boosting Recall: 73.39% Neural Network F1-score: 72% XGB AUC: 73% AdaBoost Precision: 77.8%
MIMIC-II [198]	Random Forest	Accuracy: 96% Sensitivity: 100% Specificity: 85%

^1^ Coronary artery disease; ^2^ Left ventricular end-diastolic pressure.

**Table 18:** Detecting other diseases using machine learning techniques.

Data Set/Study	Machine Learning Approach	Results
MIT-BIH RR Interval [199]	Convolutional Neural Network (CNN)	Accuracy: 81.85%
Data Hospital ECG echocardiographic dataset at New York Presbyterian Lawrence Hospital [200]	Convolutional Neural Network (CNN)	Aortic stenosis AUC-ROC: 88% Aortic regurgitation AUC-ROC: 77% Mitral regurgitation AUC-ROC: 83%
Physician-curated cohorts Stanford [201]	3D Convolutional Neural Network	Cardiac amyloidosis AUC: 83% Hypertrophic cardiomyopathy AUC: 98%
Physiobank [202]	Deep Convolutional Neural Network (ResNet)	Three-seconds ECGsignals Accuracy: 99.84% Sensitivity: 99.52% Specificity: 99.95%
In-silico ECGs [203]	1D Convolutional Neural Network	MSE: 0.2404
Careggi University Hospital [204]	Random Forest, SVM, Boosted Trees, Neural Networks, Bayesian Optimization, Random Search	Boosted Trees Accuracy: 75% F1-score: 82% AUC: 82% Neural Networks Specificity: 79% Precision: 79%
Electronic Health Record (EHR) by McKinsey Company [205]	SVM	Gender precision: 50%, Gender sensitivity: 32%, Gender F1-score: 30%, Gender accuracy: 50%, Age precision: 56%, Age sensitivity: 43%, Age F1-score: 49%, Age accuracy: 55%, Hypertension precision: 51%, Hypertension sensitivity: 52%, Hypertension F1-score: 49%, Hypertension accuracy: 51%, Heart disease precision: 49%, Heart disease sensitivity: 50%, Heart disease F1-score: 37%, Heart disease accuracy: 49%, Ever married precision: 63%, Ever married sensitivity: 6%, Ever married F1-score: 11%, Ever married accuracy: 51%, Work type precision: 52%, Work type sensitivity: 76%, Work type F1-score: 62%, Work type accuracy: 53%, Residence type precision: 51%, Residence type sensitivity: 84%, Residence type F1-score: 63%, Residence type accuracy: 51%, Glucose level precision: 51%, Glucose level sensitivity: 51%, Glucose level F1-score: 48%, Glucose level accuracy: 50%, BMI precision: 52%, BMI sensitivity: 38%, BMI F1-score: 34%, BMI accuracy: 50%, Smoking status precision: 49%, Smoking status sensitivity: 39%, Smoking status F1-score: 32%, Smoking status accuracy: 49%

## Data Availability

The data presented in this study are available on request from the corresponding author.

## Abbreviations

The following abbreviations are used in this manuscript:

CVD	Cardiovascular disease
IoT	Internet of things
IoMT	Internet of medical things
SCG	Seismo-cardiography
GCG	Gyro-cardiography
PPG	Photoplethysmography
AFib	Atrial fibrillation
ADHF	Acute descompensated heart failure
BPM	Beats per minute
SBP	Systolic blood pressure
DBP	Diastolic blood pressure
SR	Systolic rate
DR	Diastolic rate
WT	Wavelet transform
MI	Miocardial infarction
BBB	Bundle branch block
BMI	Body mass index
FDA	Food and Drug Administration

## Appendix A

In the Table A1, we present the 68 potential studies that answered the research questions described in Section 3, sorted by date.

**Table A1:** Summary of the selected proposals.

Ref.	Title	Year	Cites
[47]	Ultra-Low Power, Secure IoT Platform for Predicting Cardiovascular Diseases	2017	66
[40]	A deep learning approach for ECG-based heartbeat classification for arrhythmia detection	2018	351
[48]	Towards collaborative intelligent IoT eHealth: From device to fog, and cloud	2020	77
[49]	Noise Rejection for Wearable ECGs Using Modified Frequency Slice Wavelet 739 Transform and Convolutional Neural Networks	2019	54
[50]	An IoT patient monitoring based on fog computing and data mining: Cardiac arrhythmia usecase	2020	59
[51]	ECG signal processing and KNN classifier-based abnormality detection by VH-doctor for remote cardiac healthcare monitoring	2020	23
[52]	Designing Very Fast and Accurate Convolutional Neural Networks with Application in ICD and Smart Electrocardiograph Devices	2023	1
[53]	Improving R Peak Detection in ECG Signal Using Dynamic Mode Selected Energy and Adaptive Window Sizing Algorithm with Decision Tree Algorithm	2021	5
[54]	A Self-Contained STFT CNN for ECG Classification and Arrhythmia Detection at the Edge	2022	8
[55]	An Adaptive Cognitive Sensor Node for ECG Monitoring in the Internet of Medical Things	2021	9
[56]	One-Dimensional CNN Approach for ECG Arrhythmia Analysis in Fog-Cloud Environments	2021	39
[57]	Classification and analysis of cardiac arrhythmia based on incremental support vector regression on IOT platform	2021	9
[41]	IoT-based ECG monitoring for arrhythmia classification using Coyote Grey Wolf optimization-based deep learning CNN classifier	2022	12
[59]	Deep Cardiac Telemonitoring for Clinical Cloud Healthcare Applications	2022	-
[60]	An IoT enabled secured clinical health care framework for diagnosis of heart diseases	2023	-
[61]	Dew-based offline computing architecture for healthcare IoT	2022	8
[62]	A novel convolutional neural network structure for differential diagnosis of Wide QRS Complex Tachycardia	2023	-
[63]	Hybrid optimized convolutional neural network for efficient classification of ECG signals in healthcare monitoring	2022	5
[64]	KEdge: Fuzzy-Based Multi-AI Model Coalescence Solution for Mobile Healthcare System	2023	1
[65]	Prediction of heart abnormalities using deep learning model and wearabledevices in smart health homes	2022	6
[66]	Atrial Fibrillation Detection via Accelerometer and Gyroscope of a Smartphone	2017	130
[67]	Using PPG Signals and Wearable Devices for Atrial Fibrillation Screening	2019	49
[68]	Accurate detection of atrial fibrillation from 12-lead ECG using deep neural	2020	71
[69]	Identification of undiagnosed atrial fibrillation patients using a machine learning risk predicting algorithm and diagnostic testing (PULsE-AI): Study protocol for a randomised controlled trial	2020	9
[70]	Classification of Atrial Fibrillation and Acute Decompensated Heart Failure Using Smartphone Mechanocardiography: A Multi-label Learning Approach	2020	16
[71]	Hardware implementation of 1D-CNN architecture for ECG arrhythmia classification	2023	-
[67]	Classification of Aortic Stenosis Using Timeâ€“Frequency Features From Chest Cardio-Mechanical Signals	2019	27
[73]	Cardiac Output Estimation: Online Implementation for Left Ventricular Assist Device Support	2020	4
[74]	Revealing Unforeseen Diagnostic Image Features with Deep Learning by Detecting Cardiovascular Diseases From Apical 4-Chamber Ultrasounds	2022	-
[75]	A Wearable Sensor for Arterial Stiffness Monitoring Based on Machine Learning Algorithms	2018	23
[76]	Predicting cardiovascular events with deep learning approach in the context of the internet of things	2021	24
[77]	Bridging Nano and Body Area Networks: A Full Architecture for Cardiovascular Health Applications	2022	-
[78]	BDCaM: Big Data for Context-Aware Monitoringâ€”A Personalized Knowledge Discovery Framework for Assisted Healthcare	2016*	145
[79]	Non-invasive cuffless blood pressure and heart rate monitoring using impedance cardiography	2022	2
[80]	A Machine Learning-Empowered System for Long-Term Motion-Tolerant Wearable Monitoring of Blood Pressure and Heart Rate with Ear-ECG/PPG	2017	85
[81]	Toward Hypertension Prediction Based on PPG-Derived HRV Signals: a Feasibility Study	2018	48
[82]	Blind, Cuff-less, Calibration-Free and Continuous Blood Pressure Estimation using Optimized Inductuve Group Method of Data Handling	2022	31
[83]	Pervasive blood pressure monitoring using Photoplethysmogram (PPG) sensor	2017	48
[84]	Cuffless Continuous Blood Pressure Estimation From Pulse Morphology of Photoplethysmograms	2019	30
[85]	Continuous blood pressure measurement from one-channel electrocardiogram signal using deep-learning techniques	2020	72
[86]	Resource-Aware Mobile-Based Health Monitoring	2016	25
[87]	Smart assisted diagnosis solution with multi-sensor Holter	2017	7
[88]	Machine Learning and Mobile Health Monitoring Platforms: A Case Study on Research and Implementation Challenges	2018	11
[39]	HealthFog: An ensemble deep learning based Smart Healthcare System for Automatic Diagnosis of Heart Diseases in integrated IoT and fog computing environments	2020	432
[89]	Utilizing IoT wearable medical device for heart disease prediction using higher order Boltzmann model: A classification approach	2019	101
[90]	Wireless high-frequency NLOS monitoring system for heart disease combined with hospital and home	2020	13
[91]	Construction and Application of a Medical-Grade Wireless Monitoring System for Physiological Signals at General Wards	2020	22
[98]	Ambient assisted living predictive model for cardiovascular disease prediction using supervised learning	2021	23
[43]	An IoT Framework for Heart Disease Prediction Based on MDCNN Classifier	2020	188
[41]	A novel three-tier Internet of Things architecture with machine learning algorithm for early detection of heart disease	2018	258
[92]	Adaptive Multi-Dimensional dual attentive DCNN for detecting Cardiac Morbidities using Fused ECG-PPG Signals	2022	-
[93]	Platform for Healthcare Promotion and Cardiovascular Disease Prevention	2021	6
[94]	MedAi: A Smartwatch-Based Application Framework for the Prediction of Common Diseases Using Machine Learning	2023	1
[95]	Detection of Cardiovascular Disease Based on PPG Signals Using Machine Learning with Cloud Computing	2022	4
[96]	A Predictive Analysis of Heart Rates Using Machine Learning Techniques	2022	19
[97]	An Open-Source Privacy-Preserving Large-Scale Mobile Framework for Cardiovascular Health Monitoring and Intervention Planning with an Urban African American Population of Young Adults: User-Centered Design Approach	2022	2
[98]	Ambient assisted living predictive model for cardiovascular disease prediction using supervised learning	2021	23
[99]	An Efcient AlexNet Deep Learning Architecture for Automatic Diagnosis of Cardioâ€‘Vascular Diseases in Healthcare System	2022	5
[100]	Smart wearable model for predicting heart disease using machine learning	2022	3
[101]	Toward Real-Time, At-Home Patient Health Monitoring Using Reservoir Computing CMOS IC	2021	2
[102]	Portable and Real-Time IoT-Based Healthcare Monitoring System for Daily Medical Applications	2022	3
[103]	Dictionary Learning-Based Multichannel ECG Reconstruction Using Compressive Sensing	2022	2
[104]	Real-Time Cloud-Based Patient-Centric Monitoring Using Computational Health Systems	2022	27
[105]	A portable medical device for detecting diseases using Probabilistic Neural Network	2022	-
[106]	BeatClass: A Sustainable ECG Classification System in IoT-Based eHealth	2021	34
[107]	Non-contact Monitoring of Heart Rate Variability Using A Fiber Optic Sensor	2023	-
[108]	Remote Health Monitoring System for the Estimation of Blood Pressure, Heart Rate, and Blood Oxygen Saturation Level	2023	1
[109]	iKardo: An Intelligent ECG Device for Automatic Critical Beat Identification for Smart Healthcare	2021	6
[110]	Energy-Efficient Real-Time Heart Monitoring on Edge-Fog-Cloud Internet of Medical Things	2021	15
[111]	Real-Time Tele-Monitoring of Patients with Chronic Heart-Failure Using a Smartphone: Lessons Learned	2016	58
[112]	Fog based smart healthcare: a machine learning paradigms for IoT sector	2022	2
[113]	Real-Time Event-Driven Classification Technique for Early Detection and Prevention of Myocardial Infarction on Wearable Systems	2018	75
[114]	A High performance electronic nose system for the recognition of myocardial infarction and coronary artery diseases	2021	24
[115]	FETCH: A Deep Learning-Based Fog Computing and IoT Integrated Environment for Healthcare Monitoring and Diagnosis	2022	32
[116]	FedECG: A Federated Semi-supervised Learning Framework for Electrocardiogram Abnormalities Prediction	2023	-
[117]	Development of a PPG Sensor Array as a Wearable Device for Monitoring Cardiovascular Metrics	2021	17
[118]	AI-based stroke prediction system using body motion biosignals during walking	2022	3
[119]	A Machine Learning Pipeline for Measurement of Arterial Stiffness in A-Mode Ultrasound	2021	2
[120]	A Real-Time Tunable ECG Noise-Aware System for IoT-Enabled Devices	2022	1
[121]	Interpretable Rule Mining for Real-Time ECG Anomaly Detection in IoT Edge Sensors	2023	-
[129]	Proposition of novel classification approach and features for improved real-time arrhythmia monitoring	2016	21
[44]	A robust deep convolutional neural network with batch-weighted loss for heartbeat classification	2019	158
[38]	Arrhythmia detection using deep convolutional neural network with long duration ECG signals	2018	579
[42]	A new approach for arrhythmia classification using deep coded features and LSTM networks	2019	256
[130]	A Real-Time Arrhythmia Heartbeats Classification Algorithm Using Parallel Delta Modulations and Rotated Linear-Kernel Support Vector Machines	2019	56
[131]	Automated detection of shockable and non-shockable arrhythmia using novel wavelet-based ECG features	2019	42
[132]	Heart disease detection using hybrid of bacterial foraging and particle swarm optimization	2020	23
[133]	A Deep Biometric Recognition and Diagnosis Network with Residual Learning for Arrhythmia Screening Using Electrocardiogram Recordings	2020	3
[134]	Deep Multi-Scale Fusion Neural Network for Multi-Class Arrhythmia Detection	2020	74
[135]	Intelligent Health Vessel ABC-DE: An Electrocardiogram Cloud Computing Service	2018	17
[136]	Improving the safety of atrial fibrillation monitoring systems through human verification	2017	11
[137]	Validating the robustness of an internet of things based atrial fibrillation detection system	2020	20
[138]	Extracting deep features from short ECG signals for early atrial fibrillation detection	2020	22
[139]	Detection of Atrial Fibrillation in Compressively Sensed Electrocardiogram Measurements	2020	15
[140]	A Multi-tier Deep Learning Model for Arrhythmia Detection	2020	115
[141]	Arrhythmia classification from single-lead ECG signals using the inter-patient paradigm	2021	50
[142]	Time adaptive ECG driven cardiovascular disease detector	2021	21
[143]	ECG arrhythmia classification by using a recurrence plot and convolutional neural network	2021	91
[144]	Stacking segment-based CNN with SVM for recognition of atrial fibrillation from single-lead ECG recordings	2021	33
[145]	Exploring Deep Features and ECG Attributes to Detect Cardiac Rhythm Classes	2021	91
[146]	Automated ECG multi-class classification system based on combining deep learning features with HRV and ECG measures	2022	17
[147]	Deep learning-based multidimensional feature fusion for classification of ECG arrhythmia	2021	15
[148]	ECG signal classification to detect heart arrhythmia using ELM and CNN	2022	-
[149]	Multi-Lead ECG Classification via an Information-Based Attention Convolutional Neural Network	2020	5
[150]	Fuzz-ClustNet: Coupled fuzzy clustering and deep neural networks for Arrhythmia detection from ECG signals	2023	-
[151]	Inter-patient arrhythmia classification with improved deep residual convolutional neural network	2022	21
[152]	DeepArr: An investigative tool for arrhythmia detection using a contextual deep neural network from electrocardiograms (ECG) signals	2023	-
[153]	Two-stage detection method of supraventricular and ventricular ectopic beats based on sequential artificial features and heartbeats	2023	-
[154]	Automatic varied-length ECG classification using a lightweight DenseNet model	2023	-
[155]	Arrhythmia detection based on multi-scale fusion of hybrid deep models from single lead ECG recordings: A multicenter dataset study	2022	2
[151]	A deep learning approach to cardiovascular disease classification using empirical mode decomposition for ECG feature extraction	2023	1
[157]	Prediction of paroxysmal atrial fibrillation using new heart rate variability features	2021	23
[158]	Predicting Hypertensive Patients with Higher Risk of Developing Vascular Events Using Heart Rate Variability and Machine Learning	2020	20
[159]	Nonlinear Dynamic Modeling of Blood Pressure Waveform: Towards an Accurate Cuffless Monitoring System	2020	22
[160]	Predicting Systolic Blood Pressure in Real-Time Using Streaming Data and Deep Learning	2021	15
[161]	Cuffless blood pressure estimation based on composite neural network and graphics information	2021	10
[162]	PPG-based blood pressure estimation can benefit from scalable multi-scale fusion neural networks and multi-task learning	2022	5
[163]	A Refined Blood Pressure Estimation Model Based on Single Channel Photoplethysmography	2022	4
[164]	An advanced LAN model based on optimized feature algorithm: Towards hypertension interpretability	2021	2
[165]	Deep learning models for cuffless blood pressure monitoring from PPG signals using attention mechanism	2021	51
[166]	NABNet: A Nested Attention-guided BiConvLSTM network for a robust prediction of Blood Pressure components from reconstructed Arterial Blood Pressure waveforms using PPG and ECG signals	2023	3
[167]	DeepCNAP: A Deep Learning Approach for Continuous Noninvasive Arterial Blood Pressure Monitoring Using Photoplethysmography	2022	5
[168]	An IoT based efficient hybrid recommender system for cardiovascular disease	2019	75
[37]	Effective Heart Disease Prediction Using Hybrid Machine Learning Techniques	2019	888
[169]	An efficient IoT based patient monitoring and heart disease prediction system using Deep learning modified neural network	2020	92
[170]	HDPM: An Effective Heart Disease Prediction Model for a Clinical Decision Support System	2020	130
[45]	A Healthcare Monitoring System for the Diagnosis of Heart Disease in the IoMT Cloud Environment Using MSSO-ANFIS	2020	153
[46]	Comprenhensive electrocardiographic diagnosis based on deep learning	2020	146
[171]	An Efficient IoT-Based Platform for Remote Real-Time Cardiac Activity Monitoring	2020	35
[172]	Multi-disease big data analysis using beetle swarm optimization and an adaptive neuro-fuzzy inference system	2021	17
[173]	Cardiac disease detection using cuckoo search enabled deep belief network	2022	2
[174]	Automatic diagnosis of cardiovascular disorders by sub images of the ECG signal using multi-feature extraction methods and randomized neural network	2021	20
[175]	An intelligent heart disease prediction system based on swarm artificial neural network	2021	23
[176]	A multi-label learning prediction model for heart failure in patients with atrial fibrillation based on expert knowledge of disease duration	2023	-
[177]	WoM-based deep BiLSTM: smart disease prediction model using WoM-based deep BiLSTM classifier	2023	-
[178]	A scalable and real-time system for disease prediction using big data processing	2023	3
[179]	A novel blockchain-enabled heart disease prediction mechanism using machine learning	2022	15
[180]	Heart rate estimation network from facial videos using spatiotemporal feature image	2022	2
[181]	Predictive analysis of cardiovascular disease using gradient boosting based learning and recursive feature elimination technique	2022	3
[182]	Effective heart disease prediction using novel MLP-EBMDA approach	2022	22
[183]	LightX3ECG: A Lightweight and eXplainable Deep Learning System for 3-lead Electrocardiogram Classification	2023	2
[184]	Heart Diseases Prediction based on Stacking Classifiers Model	2023	-
[185]	Tuning Multi-Layer Perceptron by Hybridized Arithmetic Optimization Algorithm for Healthcare 4.0	2022	-
[186]	Optimized Signal Quality Assessment for Photoplethysmogram Signals Using Feature Selection	2022	8
[187]	Photoplethysmography-Based Heart Action Monitoring Using a Growing Multilayer Network	2022	-
[188]	KD-Informer: A Cuff-Less Continuous Blood Pressure Waveform Estimation Approach Based on Single Photoplethysmography	2022	6
[189]	Multiclass classification of myocardial infarction with convolutional and recurrent neural networks for portable ECG devices	2018	98
[190]	ST-Net: Synthetic ECG tracing for diagnosing various cardiovascular diseases	2020	6
[191]	Explainable Prediction of Acute Myocardial Infarction Using Machine Learning and Shapley Values	2020	47
[192]	Prognostic Value of Machine Learning in Patients with Acute Myocardial Infarction	2022	8
[193]	Efficient detection of myocardial infarction from single lead ECG signal	2021	21
[194]	Near real-time single-beat myocardial infarction detection from single-lead electrocardiogram using Long Short-Term Memory Neural Network	2021	10
[195]	Predicting cardiac disease from interactions of simultaneously-acquired hemodynamic and cardiac signals	2021	8
[196]	Multilayer perceptron based deep neural network for early detection of coronary heart disease	2021	17
[197]	Early Detection of Coronary Heart Disease using Ensemble Techniques	2021	37
[198]	Non-invasive detection of coronary artery disease from photoplethysmograph using lumped parameter modelling	2022	2
[199]	Combining Convolutional Neural Network and Distance Distribution Matrix for Identification of Congestive Heart Failure	2018	36
[200]	Deep Learning Electrocardiographic Analysis for Detection of Left-Sided Valvular Heart Disease	2022	10
[201]	High-Throughput Precision Phenotyping of Left Ventricular Hypertrophy with Cardiovascular Deep Learning	2022	32
[202]	Convolutional neural network based automatic screening tool for cardiovascular diseases using different intervals of ECG signals	2021	25
[203]	Reaction-diffusion informed approach to determine myocardial ischemia using stochastic in-silico ECGs and CNNs	2021	3
[204]	A machine learning-based risk stratification model for ventricular tachycardia and heart failure in hypertrophic cardiomyopathy	2021	19
[205]	Identifying Stroke Indicators Using Rough Sets	2020	18

## Appendix B

In the Table A2 we depict the 68 Published articles sorted by journal.

**Table A2:** Number of articles published, by journal.

Refs.	Journal	Num. of Papers
[200]	*Am. J. Cardiol.*	1
[176]	*Appl Intell*	1
[79,85,138]	*Artif Intell Med.*	3
[46]	*Artif Intell Med.*	1
[38,68,129,131,150,157,180,203,204]	*Comput Biol Med.*	9
[41,179]	*Comput Electr Eng*	2
[24,33,42,141,151,195,202]	*Comput Methods Programs Biomed*	7
[98]	*Evol Intel*	1
[132]	*Evol Syst.*	1
[44,189]	*Expert Syst. Appl*	2
[39,40,83,90]	*Future Gener Comput Syst.*	4
[196]	*Health Technol*	1
[61]	*ICT Express*	1
[37,43,45,49,52,54,55,56,80,84,94,115,133,158,169,170,191,199,205]	*IEEE Access*	18
[77,106,107,110,121]	*IEEE Internet Things J.*	5
[66,86,134,163,167,188]	*IEEE J. Biomed Health Inform*	6
[101]	*IEEE J. Emerg Sel Top Circuits*	1
[70,75,103,108,117,120,159,187]	*IEEE Sens. J.*	8
[64]	*IEEE Syst. J.*	1
[171]	*IEEE T Consum Electr*	1
[47]	*IEEE TCAS-I*	1
[111]	*IEEE Trans. Affect Comput*	1
[73,113]	*IEEE Trans. Biomed Circuits Syst.*	2
[72,130,186]	*IEEE Trans. Biomed Eng*	3
[78,135]	*IEEE Trans. Cloud Comput*	2
[102,104]	*IEEE Trans. Comput Soc Syst.*	2
[109]	*IEEE Trans. Consum*	1
[67]	*IEEE Trans. Ind Electron*	1
[139,140]	*IEEE Trans. Instrum Meas*	2
[119]	*IEEE Trans. Ultrason Ferroelectr*	1
[92]	*IEEE Trans. Artif Intell*	1
[197]	*Inform Med. Unlocked*	1
[96]	*Int. J. Environ. Res. Public Health*	1
[50,77,106,107,110,121]	*Internet Things*	6
[74]	*J. Am Heart Assoc*	1
[192]	*J. Cardiovasc. Dev. Dis.*	1
[88]	*J. Healthcare Inform. Res.*	1
[116]	*J. King Saud Univ.*	1
[81,91]	*J. Med. Syst.*	2
[69]	*J. Res. Health Sci.*	1
[149]	*Shanghai Jiaotong Univ Sci*	1
[118]	*J. Supercomput*	1
[201]	*JAMA Cardiol*	1
[97]	*JMIR Form Res*	1
[145]	*Knowl Based Syst.*	1
[85]	*Measurement*	1
[48]	*Microprocess Microsyst*	1
[160]	*Mob Netw Appl*	1
[28,65,112,148,177,178]	*Multimed Tools Appl*	6
[26,76,146,147,172,175]	*Neural Comput & Applic*	6
[87]	*Neurocomputing*	1
[137]	*Pattern Recognit. Lett.*	1
[168]	*Peer-to-Peer Netw. Appl.*	1
[59]	*Procedia Comput. Sci.*	
[136]	*Saf Sci*	1
[23,53,121]	*Sensors*	3
[51]	*Soft. Comput.*	1
[99,100]	*Wirel. Pers. Commun.*	2
